# Bursts with High and Low Load of Epileptiform Spikes Show Context-Dependent Correlations in Epileptic Mice

**DOI:** 10.1523/ENEURO.0299-18.2019

**Published:** 2019-09-05

**Authors:** Katharina Heining, Antje Kilias, Philipp Janz, Ute Häussler, Arvind Kumar, Carola A. Haas, Ulrich Egert

**Affiliations:** 1 Biomicrotechnology, Department of Microsystems Engineering - IMTEK, Faculty of Engineering, University of Freiburg, Freiburg 79110, Germany; 2 Bernstein Center Freiburg, University of Freiburg, Freiburg 79104, Germany; 3 Faculty of Biology, University of Freiburg, Freiburg 79104, Germany; 4 Experimental Epilepsy Research, Department of Neurosurgery, Medical Center, University of Freiburg, Freiburg 79106, Germany; 5 BrainLinks-BrainTools Cluster of Excellence, University of Freiburg, Freiburg 79110, Germany; 6 Computational Science and Technology, School of Electrical Engineering and Computer Science, KTH Royal Institute of Technology, Stockholm 11428, Sweden

**Keywords:** electrographic seizures, epileptic spikes, epileptiform activity, hippocampus, interictal activity, mesial temporal lobe epilepsy

## Abstract

Hypersynchronous network activity is the defining hallmark of epilepsy and manifests in a wide spectrum of phenomena, of which electrographic activity during seizures is only one extreme. The aim of this study was to differentiate between different types of epileptiform activity (EA) patterns and investigate their temporal succession and interactions. We analyzed local field potentials (LFPs) from freely behaving male mice that had received an intrahippocampal kainate injection to model mesial temporal lobe epilepsy (MTLE). Epileptiform spikes occurred in distinct bursts. Using machine learning, we derived a scale reflecting the spike load of bursts and three main burst categories that we labeled high-load, medium-load, and low-load bursts. We found that bursts of these categories were non-randomly distributed in time. High-load bursts formed clusters and were typically surrounded by transition phases with increased rates of medium-load and low-load bursts. In apparent contradiction to this, increased rates of low-load bursts were also associated with longer background phases, i.e., periods lacking high-load bursting. Furthermore, the rate of low-load bursts was more strongly correlated with the duration of background phases than the overall rate of epileptiform spikes. Our findings are consistent with the hypothesis that low-level EA could promote network stability but could also participate in transitions towards major epileptiform events, depending on the current state of the network.

## Visual Abstract

**Figure:**
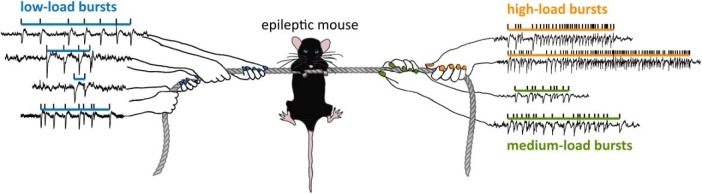

## Significance Statement

It is a major challenge in epilepsy research to understand how different types of epileptiform activity (EA) interact and whether epileptiform spikes prevent or promote major epileptic events. In our mouse model for mesial temporal lobe epilepsy (MTLE), epileptiform spikes occurred in bursts. Increased rates of bursts with low spike load were clearly associated with extended phases lacking high-load bursts, which is in line with the view that low-level activity could promote network stability in epilepsy. Low-load bursts, however, also occurred during transition phases to clusters of high-load bursts. Both findings are consistent with the hypothesis that low-level EA could reduce the excitability of the network but that its impact depends on the current state of the network.

## Introduction

Epilepsy is a disease of hypersynchronized brain activity that can be monitored electrophysiologically as epileptiform activity (EA) in local field potentials (LFP). Aside from EA accompanied by behavioral symptoms, e.g., as in clinical seizures, the EA patterns that have received the most attention thus far are epileptiform spikes and compound events termed “electrographic seizures.” The exact definition and even the existence of purely electrographic seizures, however, is being debated. There seems to be no clear boundary between electrographic seizures and other compound events, which can differ widely in spike rate and duration (Gotman, 2011). Although the evolution of spikes within larger bursts has been analyzed (Chauvière et al., 2012), a systematic quantitative time series analysis of compound events is lacking. In fact, clustering of seizures in patients (Osorio et al., 2009; Karoly et al., 2017) suggests that the incidence of other compound events might also fluctuate over time. This raises the questions whether information about the state of epileptic networks could be read from the time series of compound events and whether the occurrence of one type of EA pattern influences the occurrence of other patterns. The aim of this study was to identify different types of compound events, investigate their temporal succession and analyze interactions between different types of events in an animal model for mesial temporal lobe epilepsy (MTLE).

In MTLE, the most common form of focal epilepsy, partial seizures are frequent, while generalized convulsive seizures are rare (Engel, 2001). The intrahippocampal kainate mouse model is a well-accepted animal model to investigate MTLE because it exhibits several phenomena similar to the human condition, e.g., spatially localized EA and histologic changes (Riban et al., 2002; Guillemain et al., 2012; Häussler et al., 2012; Krook-Magnuson et al., 2013; Twele et al., 2016; Janz et al., 2017). While large behavioral seizures are as rare in this as in other rodent models of epilepsy (Cavalheiro et al., 1991; Rattka et al., 2013), events that have been labeled “electrographic seizures,” “hyperparoxysmal discharges,” or “high voltage sharp waves” recur particularly frequently in kainate-injected mice (Klee et al., 2017; Kilias et al., 2018). As detailed in Twele et al. (2016), previous studies using this model identified and classified these large EA events based on heuristic criteria and experts applied these criteria with highly divergent results. The same study demonstrated that even slight changes in event definition can have a critical impact on whether a candidate drug is found to be anti-epileptic or not. This highlights the need for a more data-driven and reproducible approach to identify different patterns of EA and their interaction during epileptiform dynamics.

Interactions between EA event types have mostly been addressed based on the incidence of individual epileptiform spikes and seizures. While specific sub-types of spikes are thought to be involved in seizure onset (De Curtis and Avoli, 2016), several studies interpreted interictal spiking as preventing seizure generation (Engel and Ackermann, 1980; Librizzi and De Curtis, 2003; Goncharova et al., 2016). Other reports proposed either that changes in interictal spike rate are governed by seizure dynamics (Jensen and Yaari, 1988; Gotman, 1991) or found the two phenomena to be independent from each other (Selvitelli et al., 2010). Recently, Chang et al. (2018) suggested that epileptiform spikes stabilize network activity in states of low excitability while the same events precipitate seizures if they occur during transition phases of excitability. Hence, temporal context should be considered when interpreting the role of EA events.

In this study, we systematically describe patterns of EA in the kainate mouse model of MTLE using a data-driven approach and investigate how such patterns interact. Motivated by the observation that spikes preferentially occurred in bursts, we developed a method that automatically transformed hippocampal LFPs into a time series of solitary spikes and bursts. These bursts were classified using machine learning techniques according to a composite measure that could best be summarized as spike load. In these time series, bursts of different categories aggregated and thus formed phases reflecting slow fluctuations of EA dynamics. We delimited these phases and found that the relationship between low-load and high-load bursting depended on temporal context. Our results suggest that the burst structure of EA plays an important role in epileptiform dynamics, that different burst patterns of EA interact and that these interactions vary over time.

## Materials and Methods

### Animals and kainate injection

Data originated from 18 adult male C57BL/6N mice (age 9–12 weeks, Charles River, Sulzfeld, Germany) that were part of previous studies and in which a detailed description of the surgical and electrophysiological procedures can be found (dataset A with *N* = 7 from Janz et al., 2017; dataset B with *N* = 12 from Froriep et al., 2012). Briefly, mice were anesthetized (ketamine hydrochloride 100 mg/kg, xylazine 5 mg/kg, atropine 0.1 mg/kg body weight, i.p.) and kainate solution (50 nl, 20 mM in 0.9% NaCl, Tocris) was injected unilaterally into the dorsal right hippocampus. After kainate injection, all mice showed symptoms of status epilepticus, such as convulsions, chewing and immobility (Riban et al., 2002; Heinrich et al., 2006). All animal procedures were in accordance with the German Animal Welfare Act and were approved by the regional council in Freiburg, Germany.

### Electrophysiological recordings

Starting two weeks after kainate injection, when recurrent EA occurred reliably, mice were re-anesthetized to implant platinum-iridium wire electrodes into both dorsal hippocampi (dataset A) or steel electrodes at the ipsilateral dorsal hippocampus and medial entorhinal cortex (dataset B). Signals were amplified 1000×, filtered at 1–5000 Hz (MPA8I and PGA32; Multi Channel Systems), and sampled at 10 kHz (Power 1401 ADC; Spike2 software, RRID: SCR_000903, Cambridge Electronic Design). Data were recorded in the molecular or granule cell layer of the dentate gyrus at 14–39 d after kainate injection from awake, freely behaving, chronically epileptic animals (three to nine sessions per mouse, each lasting 1.5–3 h, 105 sessions total). Mice from dataset A were recorded on consecutive days, mice from dataset B every other day. Large, putative behavioral seizures (compare Zeidler et al., 2018) were identified visually based on morphology and correlated EA across at least two different brain regions (dataset A: ipsilateral dentate gyrus and contralateral dentate gyrus; dataset B: ipsilateral dentate gyrus and entorhinal cortex).

### Histology

After recording was completed, mice were transcardially perfused and their brains were sectioned with a vibratome (set A: 50 μm, coronal sections; dataset B: 70 μm, sagittal sections). Sections were stained with 4′,6′-diamidino-2-phenylindole (DAPI; set A; 1:10,000; Roche Diagnostics GmbH) or cresyl violet (dataset B; as in Heinrich et al., 2006) to verify electrode positions.

### Data analysis

#### Overview

**Figure 1.**
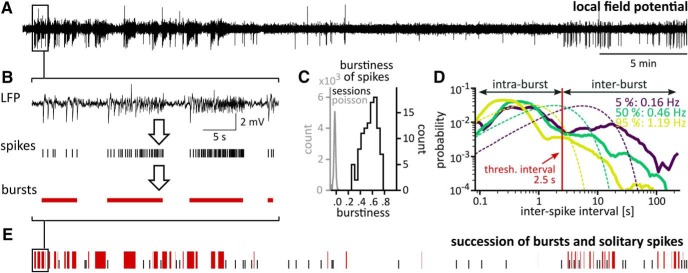
Epileptiform spikes come in bursts. ***A***, LFP recording from a kainate-treated mouse during chronic epilepsy. ***B***, Epileptiform spikes (tick marks) were detected in the LFP and used to delimit burst events (red horizontal bars). ***C***, Distribution of burstiness scores. Burstiness scores can adopt values ranging from –1 (indicating completely regular interevent intervals) over 0 (Poissonian interval distribution) to 1 (extreme burstiness of events). Spike series in the recording sessions (black, *N* = 105) have distinctly higher burstiness than their Poissonian surrogates (gray, *N* = 105,000). ***D***, Defining bursts. Interspike intervals from three sessions representing the 5th (deep purple), 50th (green), and 95th (yellow) percentile of spike rates. Spike rates are displayed next to the percentiles. We interpreted the location of the valley/plateau in these distributions as separating short intraburst intervals from long interburst intervals and grouped spikes closer than 2.5 s (red vertical line) into the same burst event. Interspike interval distributions of surrogates generated with matching spike rates are shown as dotted lines in the color of their reference recording. ***E***, We thus converted continuous LFP patterns into time series of discrete EA events: bursts (red) and solitary spikes (black ticks). Extended Data Figure 1-1 compares the succession of spikes and bursts to a rate profile generated from spike timings and shows that bursts match distinct peaks in the rate profile.

**Figure 2.**
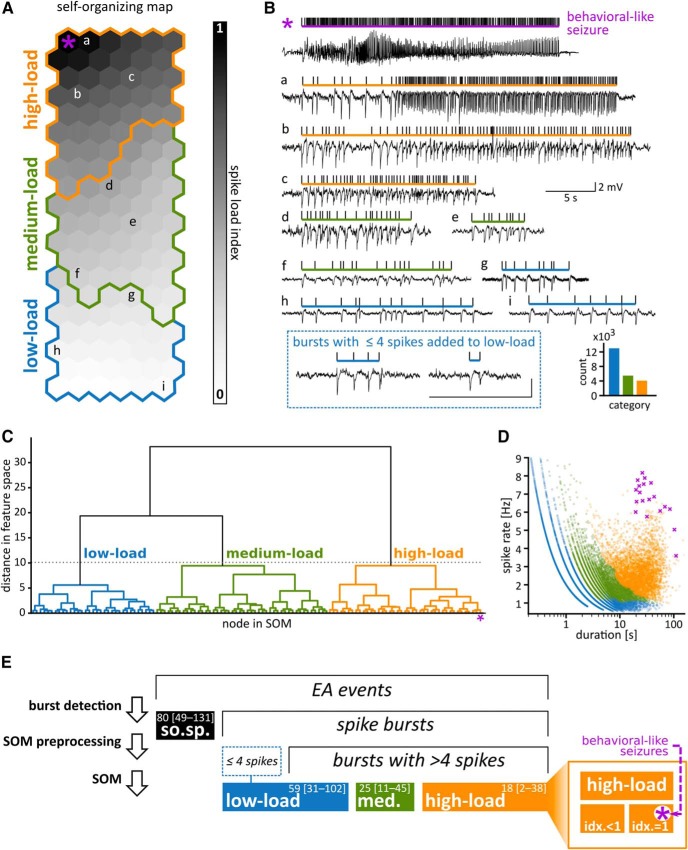
Classification of epileptic bursts. ***A***, A SOM was used to transform burst features (number of spikes in a burst, median interspike interval, SD of interspike intervals) into a spike load index. Each node (hexagon) represents a combination of features. Bursts were assigned to nodes that match their features best (letters refer to the examples shown in ***B***). The spike load index (gray scale) captures the proximity to the node containing the bursts with the highest spike loads (*). Colored lines enclose nodes grouped into categories by a dendrogram analysis (see ***C***). ***B***, Examples of EA events after classification. Vertical ticks above the LFP traces mark individual spikes found by our detection algorithm. Horizontal bars delimit the extent of spike bursts, with color indicating the category assigned to the burst. Lower case letters (a–i) refer to their placement on the SOM. Bursts with less than five spikes were not classified on the SOM and later added to the category low-load bursts. The inset shows how many bursts of each category were present in the dataset. Participation of the contralateral DG and ipsilateral EC varied for bursts of different categories (Extended Data Figure 2-2). ***C***, Nodes of the SOM were hierarchically clustered using Ward’s dendrogram method (Ward, 1963). To create the dendrogram, nodes were successively merged according to their proximity in feature space. The *y*-axis shows the distance in feature space between merges. The gray dotted line indicates the threshold used to obtain the three categories as suggested by Thorndike’s criterion (Extended Data Figure 2-1). ***D***, Spike rate against duration of bursts. Each dot marks one burst and is colored according to its category. Bursts of the same duration or spike rate can belong to different categories. Purple crosses mark visually identified, putative behavioral seizures. ***E***, Overview of terminology and event rates. The population of EA events had been split into spike bursts and solitary spikes (so.sp.) by burst detection (Fig. 1). Bursts with more than four spikes were classified using a SOM (***A***) and subsequent hierarchical clustering (***C***), resulting in three main categories. The category high-load bursts included events with the highest spike load index (idx. = 1; inset). Seizures that had been identified visually beforehand all scored spike load index = 1 in the SOM. Numbers indicate median and 10th–90th percentile range of event counts per hour across sessions (*N* = 105).

**Figure 3.**
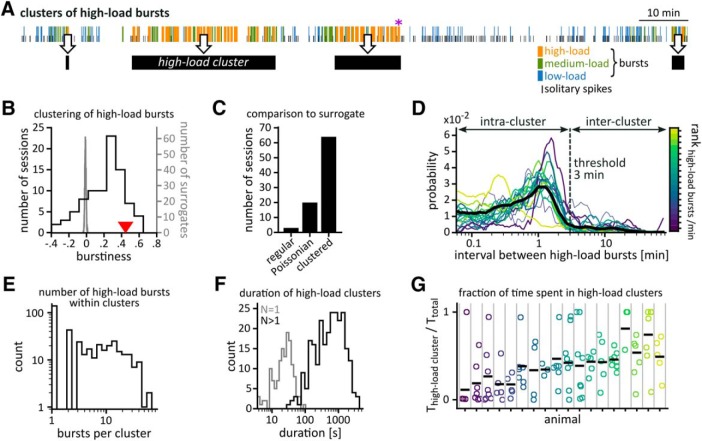
Clusters of high-load bursts. ***A***, Sample sequence of classified EA events. The purple asterisk marks a putative behavioral seizure. Black bars below the time series indicate high-load clusters defined according to ***D***. Our definition allowed for high-load clusters to consist of a single high-load burst. ***B***, Burstiness calculated from the intervals between all high-load bursts in the full dataset (value marked by red triangle) and for each session separately (distribution shown in black, *N* = 87, including only sessions with at least five intervals between high-load burst). The distribution of surrogate burstiness scores (gray) was derived from surrogate series of high-load bursts matching the rate of high-load bursts in the whole dataset (*N* = 1000). ***C***, Number of sessions scoring burstiness values significantly below (“regular”), significantly above (“clustered”), or not significantly different from surrogate interval distributions. For each recording, we generated 1000 surrogate interval distributions of matching rate and compared their burstiness distribution to the original data. Significance level: α ≤ 0.05. ***D***, The distributions of intervals between high-load bursts were used to define clusters of high-load bursts. Colored lines indicate distributions for individual animals; the average distribution across animals is shown in black. High-load bursts separated by <3 min (vertical line) were grouped into the same high-load cluster. ***E***, Distribution of cluster size, i.e., the number of high-load bursts within high-load clusters. ***F***, Distribution of duration of high-load clusters. Gray, high-load clusters consisting of a single high-load burst; black, clusters containing more than one high-load burst. ***G***, Fraction of time spent in high-load clusters for all sessions (circles, colors as in ***D***). Horizontal dashes indicate medians across recording sessions of the same mouse (separated by vertical lines).

**Figure 4.**
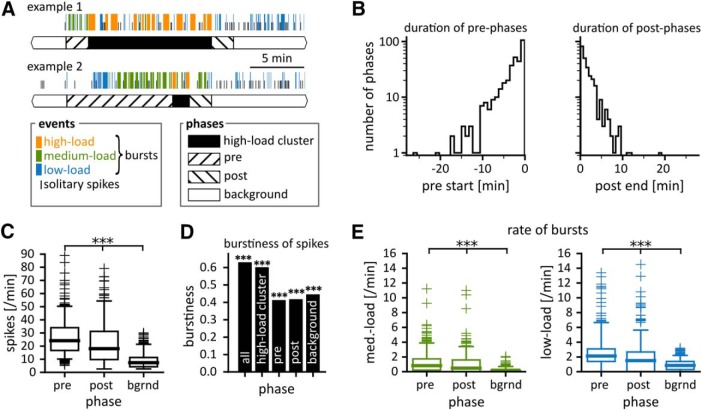
Transition phases surround high-load clusters. ***A***, Two example sequences with increases of low-load and medium-load bursts during transitions before (“pre”) and after (“post”) high-load clusters. Background phases (white bars) were defined by exclusion (neither high-load cluster nor transition phase, see Extended Data Fig. 4-1 for the step-wise definition of these phases). Post-phases could also consist of a depression of EA that was sometimes followed by a rebound of EA (Extended Data Fig. 4-2). ***B***, Distributions of the start of pre-phases (left) and end of post-phases (right) with respect to the onset (left), respectively, offset (right) of high-load clusters. These distributions are equivalent to the duration of the phases. Note that the *y*-axis is scaled logarithmically. ***C***, Spike rates for pre-phases (*N* = 347), post-phases (*N* = 311), and background phases (*N* = 340).^b^ Within each box the middle bar indicates the median, while the lower and upper boundaries mark the 25th and 75th percentiles, respectively. Whiskers extend 1.5 times the range from the 25th to 75th percentiles. Crosses show data points outside of the whisker range. ***D***, Burstiness of spikes in all phases combined, i.e., the whole dataset (“whole”) and calculated for each type of phase separately. Significance was derived by comparison to 1000 spike trains with interspike intervals generated from a Poissonian distribution and matching rate. All phases scored burstiness values higher than any of their Poissonian surrogates (***p < 0.001). ***E***, Rates of medium-load (left) and low-load (right) bursts in pre-phases, post-phases, and background phases.^c,d^ Statistics: Kruskal–Wallis test for differences across groups (*p* < 0.001; ***C***, ***E***), followed by pairwise Mann–Whitney test with Bonferroni correction (***p < 0.001; ***C***, ***E***).

We delimited bursts of epileptiform spikes as EA events (Fig. 1) and classified them according to spike load (Fig. 2) using a machine learning algorithm. From the succession of classified EA events we derived phases of larger temporal scale (Figs. 3, 4; workflow in Extended Data Fig. 4-1*A*). LFPs obtained from ipsilateral septal recording sites were analyzed in detail. The first 10 min of each recording were discarded to avoid residual influence of the brief isoflurane anesthesia applied to connect the headstage. Periods with movement or other recording artifacts were identified visually and masked before further analysis.

10.1523/ENEURO.0299-18.2019.ed1 Extended Data Figure 1-1 Comparison between rate profile and detected bursts. ***A***, top, Sequence of epileptiform spikes detected in the LFP shown in Figure 1*A*. Middle, By delimiting spike bursts (Fig. 1*D*), we derived time series of solitary spikes (black tick marks) and bursts (colored rectangles). Bursts are colored according to category as detailed in Figure 2. The purple asterisk marks a visually identified, putative behavioral seizure. Bottom, Rate profile generated by convolving the spike times (top) with a Gaussian kernel (width: 5 s, SD: 1.66 s). Generating rate profiles with other kernel shapes and widths yielded similar results. Zooming (***B–D***) reveals that detected bursts coincide with separable peaks or chains of peaks (e.g., ***D***, last high-load burst) in the rate profile. This corroborates the notion that the bursts detected by our time series approach are indeed distinct events. Extracting such events from a rate profile would have yielded similar results but would have required heuristic tuning of more parameters. Download Extended Data Fogire 1-1, TIF file.

10.1523/ENEURO.0299-18.2019.f2-1 Extended Data Figure 2-1 Determining a suitable number of burst categories. A dendrogram analysis was used to create a hierarchy of similarity between nodes of the SOM (Fig. 2*A*,*C*). We used Thorndike’s criterion (Thorndike, 1953) to determine a suitable number of categories, i.e., main branches of the dendrogram. The graph shows the distance in feature space between the last pair merged as a function of the number of categories defined. Following Thorndike, we chose three categories (gray dotted line) as the curve flattens from three categories onwards. Download Figure 2-1, TIF file.

10.1523/ENEURO.0299-18.2019.ed1 Extended Data Figure 2-2. Representative EA from the kainate-injected dentate gyrus, the contralateral dentate gyrus, and the entorhinal cortex. ***A***, Visually identified, putative behavioral seizures in the ipsilateral dentate gyrus (DG-ipsi, top traces) were accompanied by strong high-frequency and high-amplitude activity on the contralateral side (DG-contra, left), and generalized to the entorhinal cortex (EC-ipsi, right). Horizontal bars delimit automatically detected ipsilateral events and are colored according to the category assigned. Visually identified seizures scored spike load index 1. ***B***, Other bursts with spike load index = 1 were reliably accompanied by intense contralateral EA, but only little synchronous activity in the entorhinal cortex. ***C***, Likewise, ipsilateral high-load bursts with spike load index of <1 often had synchronous EA in the contralateral dentate gyrus with sparser spiking in the entorhinal cortex. ***D***, Medium-load and low-load bursts were morphologically similar to trains of spike-and-wave complexes. During low-load bursts the wave-component was salient, while during medium-load bursts spike patterns typically became denser as spike amplitudes decreased and wave-components appeared less prominent. During medium-load bursts, we typically observed contralateral spiking and sparse spiking could also occur in the entorhinal cortex. Scale bars: 20 s, 1 mV. DG-ipsi/DG-contra pairs (left column) originate from dataset A, DG-ipsi/EC-ipsi pairs (right column) from dataset B. Download Extended Data Figure 2-2, TIF file.

10.1523/ENEURO.0299-18.2019.f4-1 Extended Data Figure 4-1 Step-wise definition of high-load clusters, transition phases and background phases. ***A***, High-load clusters, transition and background phases were derived from the sequence of classified bursts (rectangles, colored according to category). □ High-load bursts closer than 3 min were grouped into the same high-load cluster (black boxes, see Fig. 3). □ Regions rich in medium-load and low-load bursts around high-load clusters were delimited (pre and post, hatched) using change-point analysis. Post-phases could also consist of long burst-free intervals following high-load clusters (“depression,” see ***B*** and Extended Data Fig. 4-2*A*). In a few cases, short lapses between two high-load clusters were occupied by dense clusters of medium-load bursts (“pre|post”). These were identified based on the distribution shown in ***C***. □ + □ From the remaining epochs, we only defined periods longer than 90 s as background phases (***C***). ***B***, Cumulative interburst interval distribution within periods that neither belonged to high-load clusters nor to pre-phases, nor to post-phases rich in bursts (*N*_intervals_ = 6622). A threshold of 112 s (95th percentile of these intervals) was used to identify unusually long intervals following a high-load cluster as depression. ***C***, Fraction of time occupied by medium-load bursts in a period vs. duration of that period shown for periods selected as in ***B***, excluding depression periods. Pre|post indicates periods with more than 15% of time occupied by medium-load bursts. Periods shorter than 90 s were defined as short. ***D***, Occurrence of the different phases in the full dataset. High-load clusters were most abundant, while short and pre|post phases were rare. ***E***, Total time spent in each phase. High-load clusters and background phases each accounted for about 40%. Download Figure 4-1, TIF file.

10.1523/ENEURO.0299-18.2019.f4-2 Extended Data Figure 4-2 The dual nature of the post-phase. ***A***, Two examples of periods following high-load clusters. Sequences of EA events are shown above, the phases derived from them are shown below. A high-load cluster could be followed by an episode without bursts terminated by a cluster of bursts (“rebound”). The second example shows a period in which a high-load cluster ended in a visually identified seizure and was followed by a long period without bursts and without obvious rebound (“depression”). Δ denotes the delay between the end of the last burst, which must be a high-load burst by definition, in a high-load cluster and the start of the first following burst. ***B***, Correlation between spike load index of the last burst in a high-load cluster and delay Δ (*N* = 409, τ = 0.28, *p*_τ_ = 8.5 × 10^−18^). Blue crosses mark the values obtained for visually identified seizures. High-load clusters ending with bursts of higher spike load are followed by longer delays. Download Figure 4-2, TIF file.

#### Detecting epileptiform spikes in the LFP

LFPs were downsampled to 500 Hz. We then calculated spectrograms (fast Fourier transform, 256 ms sliding windows, normalized per frequency bin) and averaged the spectrograms in the 4–40 Hz band. We detected peaks in this activity band as epileptiform spikes. The dead time before a new spike could be detected was 0.0833 s. In addition, we identified slow high-amplitude deflections that escaped the spectrogram-based detection. Such peaks were detected if they crossed a threshold of ±4.5× the SD of the LFP baseline, which consisted of episodes where no peaks had been detected in the spectrogram. Cutouts around detections were then sorted according to their waveform by principal component analysis (five clusters identified by a Gaussian mixture model on the first three components, separately for positive and negative spikes). Events in the cluster with the lowest average peak-to-peak amplitude did not conform to the typical morphology of epileptiform spikes and were discarded. Overlapping peaks within dense bursts were directly included into the pool of spikes without prior sorting.

The spike detection algorithm was validated by a human expert who manually marked spikes in 197 randomly selected 20-s epochs (1550 spikes in total). Automatically detected spikes that occurred within a 150-ms tolerance window around the manually selected spikes were considered as true positives (TPs) and spikes outside this window as false positives (FPs). Manually selected spikes without corresponding automatically detected spikes within the tolerance window were considered false negatives (FNs). The spike detection algorithm had a sensitivity score [TP/(TP+FN)] of 0.87 and a precision score [TP/(TP+FP)] of 0.90. The average FP rate was 1.9 FP/min.

#### Delimiting bursts

A popular method to delimit bursts is based on interspike interval distributions: When these distributions are bimodal, the valley can be used to separate short intraburst intervals from long interburst intervals (Selinger et al., 2007). The interspike interval distributions of our recording sessions showed valleys or plateaus at around 2.5 s (Fig. 1*D*). We therefore used this value as a threshold to delimit spike bursts. Spikes closer than 2.5 s were grouped into the same event. Bursts closer than 3.5 s were joined. Our dataset contained 22,578 bursts and 17,814 solitary spikes.

#### Classification of bursts

To classify bursts and derive a measure of spike load we used a self-organizing map (SOM; Kohonen, 2013). A SOM can transform a high-dimensional feature space into a two-dimensional map of nodes such that neighboring nodes represent similar feature combinations. The map thus creates a, not necessarily linear, continuous representation of the dataset in feature space that can be readily visualized and interpreted.

##### Feature selection and SOM training

To develop a measure of spike load, we exclusively used features associated with the time series of spikes contained in a burst. Since subtle differences in electrode placement and recording quality can affect signal amplitude, we did not use measures related to LFP amplitude. The feature vectors contained values of the following weighted quantities for each burst: [log(number of spikes) × 2; log(median interspike interval) × 2; (SD of interspike interval) × 1]. Feature values were *z*-scored across all bursts from all animals and sessions (*N* = 12,373) and then used to train the SOM with the batch algorithm provided by Carrasco Kind and Brunner (2014)

##### Spike load index

The node representing the highest spike count and lowest median interspike interval (H*) was located at the top left. For each node, we calculated the Euclidean distance between its feature vector and the vector of H*. We then converted these distances to values between 0 and 1 to define a spike load index, with spike load index = 1 assigned to H*.

##### Categories

To identify groups of nodes with similar features we hierarchically clustered the feature vectors corresponding to the nodes (Fig. 2*C*) and selected an appropriate number of categories according to the Thorndike criterion (Thorndike, 1953; Extended Data Fig. 2-1). The dendrogram was constructed using Ward’s method (Ward, 1963) implemented in the Python package scipy.cluster.hierarchy (version: 0.17.0). We thus derived three categories and labeled them according to the average spike load index of their nodes “high-load,” “medium-load,” and “low-load.”

##### Re-mapping spike load index and categories to bursts

Each burst inherited cluster affiliation and spike load index from its best matching node, i.e., the node of the SOM whose features were most similar to the features of the burst (criterion: smallest Euclidean distance in feature space). High-load and low-load bursts gathered in opposite regions of the SOM (Fig. 2*A*,*B*).

##### Bursts with less than five spikes

Bursts with less than five spikes were not used to train the SOM because preliminary tests had shown that they would disrupt the continuous representation of the map due to their high internal variability with respect to the features used. Across sessions, the rates of burst with less than five spikes strongly correlated with the rates of low-load bursts (*N* = 105, Kendall’s τ = 0.43, *p*_τ_ = 6.6 × 10^−11^) and they were therefore assigned to the low-load category (Fig. 2*B*,*E*).

#### Defining phases

Transforming the LFP into a sequence of classified EA events and silent periods allowed us to discern their patterns on a larger scale. In a series of processing steps, we identified and delimited three main temporal phases (Extended Data Fig. 4-1*A*): (1) clusters of high-load bursts, (2) transition phases around these high-load clusters, and finally (3) background phases. The burst categories (high-load, medium-load, and low-load bursts) used to define these phases are described in detail in the results section.

##### High-load clusters

As high-load bursts clustered temporally (Fig. 3*A–C*), we defined the temporal extent of these aggregations as high-load clusters. To delimit high-load clusters, we analyzed the distribution of intervals between high-load bursts. At 3 min, the peaks of these distributions had leveled off (Fig. 3*D*). Hence, high-load bursts closer than 3 min were grouped into the same high-load cluster. A high-load cluster started with the onset of its first high-load burst and ended with the offset of its last high-load burst. High-load clusters could consist of individual high-load bursts.

##### Transition phases with increased burst rate

We observed that the probabilities for low-load and medium-load bursts were increased around high-load clusters. To detect putative transition phases we z-scored the intervals between bursts and applied the CUSUM algorithm (Gustafsson, 2001) to identify change points in the interburst interval sequence between two high-load clusters (threshold: cumulative sum of change larger than 1.5; drift compensation: 0.1). We then selected the last change point toward shorter intervals before a high-load cluster to delimit the beginning of the pre-phase. Similarly, the first change point toward longer intervals following a high-load cluster was defined as the end of the post-phase. Transition phases were not assigned when (1) the CUSUM algorithm did not detect a change point or when (2) the fraction of time spent in bursts during a candidate transition phase was less than the fraction of time occupied by such events in the remaining unassigned portion of the recording session. We thereby ensured that bursting in transition phases was above the average of the session excluding high-load clusters.

##### Transitions with depression of bursting after high-load clusters

Some high-load clusters were followed by extended burst-free periods, reminiscent of post-ictal depression. These periods could be components of the transitions from high-load clusters. To identify depression periods, we pooled interburst intervals from unassigned periods of all recordings. We assigned burst-free periods longer than the 95th percentile (112 s) of the interburst interval distribution to post-phases (Extended Data Fig. 4-1*B*).

##### Exclusion of session borders and regions around artifacts

To avoid potential contributions of ongoing post-phases at the beginning of a recording to the analyses of background phases, we excluded the first 3 min of each recording (following the 10 min excluded to avoid the effects of anesthesia) unless a high-load cluster ended in this window. To avoid contributions of putative pre-phases ending after the recording, we likewise excluded the last 5 min of a session from background phases. These settings were derived from the distributions of pre-phase and post-phase durations (Fig. 4*B*). We further excluded phases rendered ambiguous by movement-induced artifacts.

##### Background phases

**Figure 5.**
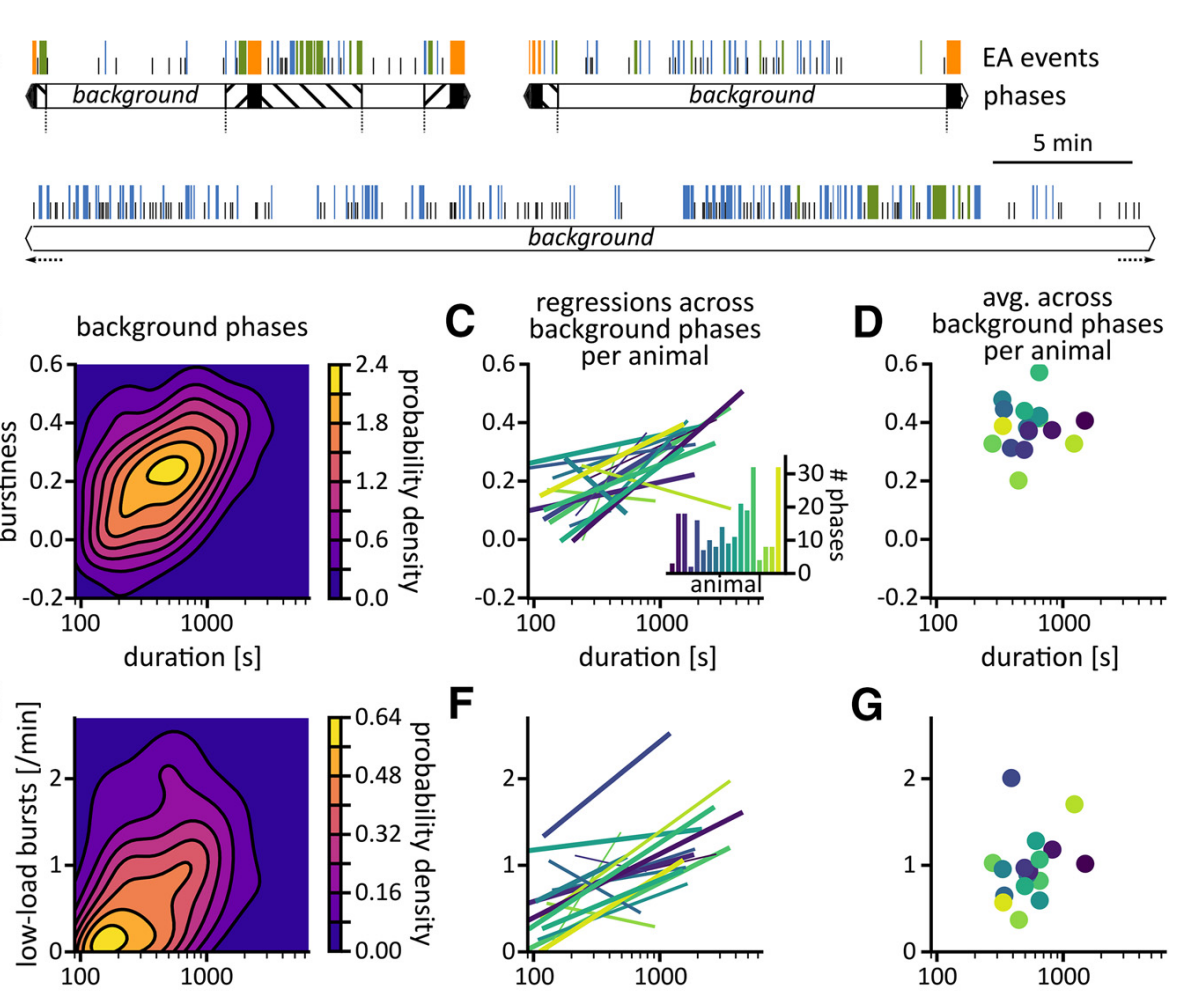
Background phases with a high rate of low-load bursts last longer. ***A***, Example sequences of EA events (colored bars: bursts; solitary spikes: black tick marks) and background phases (white boxes). ***B***, Burstiness of spikes within a background phases against duration of that background phase. Only background phases of known duration and with at least five interspike intervals were included (*N* = 197, τ = 0.27, *p*_surrogate_ = 0.009). ***C***, Relation between duration and burstiness of background phases per animal. Each line shows the least-squares regression across background phases for each animal individually (same data as in ***E*** but analyzed per animal). The slopes were positive in 15 out of 18 animals. Line width indicates the number of background phases (thin: *N* < 5, medium: *N* = 5–10, thick: *N* > 10). Inset: Number of background phases per animal. ***D***, Overall burstiness plotted against average duration of background phases per animal. The correlation was not significant (*N* = 18, τ = 0.07, *p*_surrogate_ = 0.3). Note that burstiness was calculated from interspike intervals of all background phases pertaining to an animal. Hence background phases with more intervals had a greater influence on overall burstiness of an animal. Because longer background phases, i.e., those with more intervals and/or higher spike rates, scored higher burstiness, the center of mass of the burstiness distribution per animal is shifted toward higher burstiness values compared to the center of mass of the distribution per background phase shown in ***B***. ***E***, Correlation between duration of background phase and rate of low-load bursts during background phase. Only background phases of known duration were included (*N* = 240, τ = 0.28, *p*_surrogate_ < 0.001). ***F***, Relation between background rate of low-load bursts and duration of background phase per animal. Each line shows the least-squares regression across background phases for each animal individually (same data as in ***E*** but analyzed per animal). ***G***, Correlation between average duration of background phase and average background rate of low-load bursts per animal (*N* = 18, τ = 0.20, *p*_surrogate_ = 0.5). The slopes were positive in 15 out of 18 animals. The fraction of time spent in background phases per session/animal and the survival function of background phases are shown in Extended Data Figure 5-1. Surrogate tests based on Poissonian event trains for the relationships across background phases (***B***, ***E***) and permutation tests for the relationships calculated across the set of animals (***D***, ***G***) are shown in Extended Data Figure 5-2. In Extended Data Figure 5-3, we compared the correlation shown for low-load bursts (***E***) with correlations for spikes and solitary spikes and assessed the robustness of these correlations against changes in burst definition.

To avoid misidentification of short lapses between high-load dynamics as background activity, we excluded periods <90 s (“short”) from background phases (Extended Data Fig. 4-1*C*). Since medium-load bursts were strongly associated with transition dynamics, we further excluded periods with >15% of their time occupied by medium-load bursts (Extended Data Fig. 4-1*C*). All remaining periods not classified as any of the above were considered background phases. Recording time was spent mostly in background phases (43%) and high-load clusters (40%; Extended Data Fig. 4-1*E*). Only complete high-load clusters were used to calculate the statistics of high-load clusters (data presented in Fig. 3*E*,*F*). Likewise, only data of background phases of known duration is shown in Figure 5*B–G*.

10.1523/ENEURO.0299-18.2019.f5-1 Extended Data Figure 5-1 Fraction of time spent in background phases and survival function. ***A***, Fraction of time spent in background phases for all sessions sorted according to animal. Horizontal dashes indicate medians across recording sessions of the same mouse. ***B***, Survival functions of background phases from all mice individually (colors) and across mice (black). The survival function shows what fraction of background phases (*y*-axis) lasts for at least a certain time (*x*-axis). Download Figure 5-1, TIF file.

10.1523/ENEURO.0299-18.2019.f5-2 Extended Data Figure 5-2 Surrogate and permutation tests for correlations in Figure 5. ***A***, Test for significance of the correlation between burstiness of spikes and the duration of background phases. The null hypothesis was that the observed correlation (left panel; repeated from Fig. 5*B*) was due to a continuous random process in the background generating spikes with fixed rate. Surrogate spike trains were generated from a Poissonian distribution of intervals to match the average spike rate across background phases. Surrogate correlations were obtained by computing the correlation for surrogate background phases with durations as in ***A***, left, populated by surrogate spike trains. To assess significance, we compared Kendall’s tau of the original data (τ = 0.27, red triangle in right panel) to a distribution of Kendall’s taus obtained from 1000 surrogate correlations. *p*_surrogate_ was defined as the fraction of surrogate correlations exceeding the original correlation. The burstiness of the original spikes was significantly correlated with the duration of background phases (*p*_surrogate_ = 0.009). ***B***, Using a permutation test, we assessed whether animals with on average longer background phases had spike activity with generally higher burstiness (left: original data; repeated from Fig. 5*D*). We generated surrogate data by randomly allocating background phases from the overall pool such that each animal was assigned as many background phases as it originally contributed to the dataset. The corresponding burstiness of a resulting surrogate animal was calculated from all interspike intervals in all background phases assigned to it. Significance was assessed by comparing the original correlation (τ = 0.07) to 1000 correlations from surrogate animals. Overall burstiness of spikes in background phases from an animal did not indicate its grand average duration of background phases (left-tailed *p*_surrogate_ = 0.3). ***C***, Significance of the correlation between the background rate of low-load bursts and the duration of background phases (left panel; repeated from Fig. 5*E*). We generated surrogate background phases from time series of low-load bursts with durations drawn from the overall distribution of low-load bursts in all background phases, separated by interburst intervals drawn from a Poissonian distribution. The rate parameter of the Poissonian distribution was set to yield the average rate of low-load bursts across all background phases. Significance was assessed by comparing Kendall’s tau of the original data (τ = 0.28, red triangle in right panel) to a distribution of Kendall’s taus obtained from 1000 surrogate correlations. The correlation between the background rate of low-load bursts and the duration of background phases was significant (right-tailed *p*_surrogate_ < 0.001). ***D***, Permutation test for the correlation between average background rate of low-load bursts and the average duration of background phases (left; repeated from Fig. 5*G*) across the set of animals. The grand average duration of background phases in an animal did not indicate its rate of low-load bursts averaged across these phases (*p*_surrogate_ = 0.5). Download Figure 5-2, TIF file.

**Figure 6:**
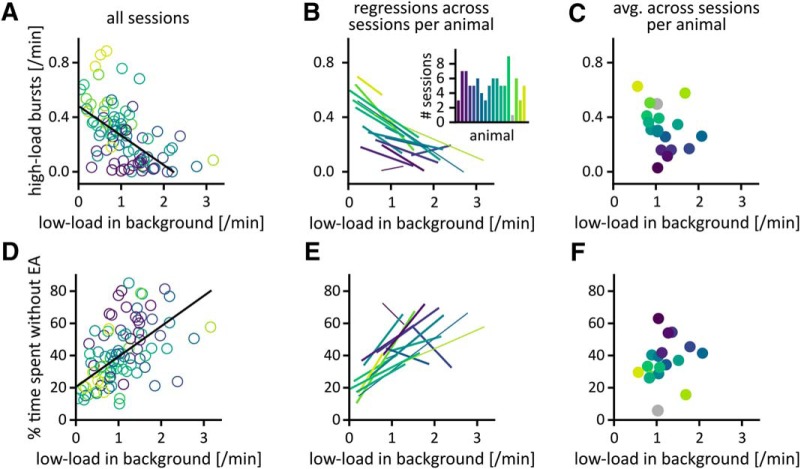
High background rates of low-load bursts, reduced susceptibility to high-load bursts. ***A***, Correlation across sessions between the average rate of low-load bursts during background phases and the rate of high-load bursts. Only sessions with background phases were included (*N* = 91, τ = –0.43, *p*_surrogate_ < 0.001). ***B***, Relation between background rate of low-load bursts and rate of high-load bursts per animal. Each line shows the least-squares regression across all sessions of each animal individually (same data as in ***A*** but analyzed per animal). The slopes were negative in 15 out of 17 animals. Line width indicates the number of background phases (thin: *N* < 5, thick: *N* ≥ 5). ***C***, Correlation between the average background rate of low-load bursts and the average rate of high-load bursts per animal (*N* = 18, τ = –0.33, *p*_surrogate_ = 0.31). The gray dot shows data for an animal that had only one session containing background phases. ***D***, Correlation across sessions between the average rate of low-load bursts during background phases and the percentage of time spent without EA (*N* = 91, τ = 0.39, *p*_surrogate_ < 0.001). EA-free episodes were defined as periods within the background phase lacking EA events. Long EA-free episodes occurred throughout the extent of interictal phases (Extended Data Fig. 6-1).^j^ ***E***, Relation between background rate of low-load bursts and percentage of time spent without EA per animal. Each line shows the least-squares regression across all sessions of an animal (same data as in ***D*** but analyzed per animal). The slopes were positive in 14 out of 17 animals. ***F***, Correlation between the average background rate of low-load bursts and the total percentage of time spent without EA per animal (*N* = 18, τ = 0.29, *p*_surrogate_ = 0.32). Surrogate tests based on Poissonian event trains for the relationships across sessions (***A***, ***D***) and permutation tests for the relationships across the set of animals (***C***, ***F***) are shown in Extended Data Figure 6-2. In Extended Data Figure 6-3, we compare our results to simulations of rate-modulated spiking processes.

10.1523/ENEURO.0299-18.2019.f5-3 Extended Data Figure 5-3 Correlation effects of low-load bursts are robust against changes in burst definition. To test how robust our results were to variations in event definition, we repeated our analyses for different burst delimitation thresholds. Thus far, we had grouped spikes separated by less than 2.5 s into the same burst event. Depending on the choice of this threshold, individual spikes could be assigned to different event types and the delineation of high-load clusters, transition and background phases could change as a consequence. To test the impact of such changes in event delimitation, we varied the threshold between 1.5 and 5 s and recalculated the correlations shown in Figures 5*E* and 6*A*,*D* for low-load bursts, solitary spikes, and spikes in general. ***A***, Illustration of EA in a background phase. “All spikes” comprises solitary spikes as well as those constituting low-load (blue) and medium-load bursts (green). ***B***, Interspike interval distributions from three sessions closest to the 5th (deep purple), 50th (i.e., median, green), and 95th percentile (yellow) of total spike rate across sessions. Thresholds for burst identification were varied from 1.5 to 5 s (gray lines, black: 2.5 s corresponding to the original threshold used as detailed in Fig. 1*D*). ***C***, Across all thresholds for burst delimitation, the duration of the background phase correlated most strongly with the rate of low-load bursts. Black vertical line, Interval threshold of 2.5 s. Blue arrow, Relation shown in Figure 5*E*. ***D***, *p* values corresponding to the correlations shown in ***C***. Red lines mark *p_τ_ < 0.05, **p_τ_ < 0.01, ***p_τ_ < 0.001 significance levels. ***E***, Of all markers, the background rate of low-load bursts was most strongly anti-correlated to the rate of high-load bursts. Blue arrow, Relation shown in Figure 6*A*. ***F***, *p* values corresponding to the correlations shown in ***E***. ***G***, Across all thresholds, the background rate of low-load bursts yielded the highest correlation to the percentage of time spent without EA. Blue arrow, Relation shown in Figure 6*D*. ***H***, *p* values corresponding to the correlations shown in ***G***. Download Figure 5-3, TIF file.

### Code accessibility

Data analysis was conducted using a custom algorithm developed in Python (optimized for versions 2.7 and 3.4) on Linux and Windows machines. The code for detecting and classifying EA and delimiting phases is available on reasonable request. The Python code for simulating rate-modulated spike trains (Extended Data Fig. 6-3) is freely available at doi:10.5281/zenodo.3376778.

### Statistics

Statistical tests and data analyses were performed with Python 2.7 (RRID: SCR_008394, Python Software Foundation). Since most variables were not normally distributed, statistical dependence was assessed using the non-parametric correlation coefficient Kendall’s tau (τ, *p* value indicated as *p*_τ_) in all cases. Correspondingly, regression lines show the Theil-Sen estimator (median of slopes through all pairs of sample points), unless stated otherwise. Burstiness was defined as $\frac{standarddeviation-mean}{standarddeviation+mean}$ of interevent intervals (Goh and Barabási, 2008). Survival functions were calculated according to the Kaplan–Meier method (Lifelines Python package, version: 0.9.4, doi:10.5281/zenodo.805993). Differences between groups were tested with the Kruskal–Wallis test. Differences between pairs of independent groups were tested by a Mann–Whitney *U* test. For pairwise comparison of dependent samples, we used Wilcoxon signed-rank test; *p* values were corrected for multiple testing with the Bonferroni method. A one-sample *t*-test was used to assess whether the distribution of fitted regression slopes significantly deviated from a random population with mean = 0. Statistical power was assessed with G*Power 3.1 (Faul et al., 2009). Observed power and exact *p* values are reported in Table 1. Datasets A and B yielded comparable results throughout this study and were therefore pooled. All value ranges reported in this study are given as the 10th–90th percentile. Per-session background rate of events was calculated by counting events within all background phases of a session and dividing by the total time spent in background phases in a session. We defined the rate of high-load bursts as the count of all high-load bursts in a session divided by the total duration of that session excluding session borders and periods spent with movement artifacts. Averages per animal were computed as weighted averages, with duration of background phase (Fig. 5*D*,*G*) or time spent in background phases in a session (Fig. 6*C*,*F*) as a weighting factor.

**Table 1.** Power analyses and exact *p* values

	Reference	test	Sample size	Observed power (α = 0.05)	*p* value
a	Text	ANOVA	a.m: 53 p.m: 52	0.15	0.65
b	Fig. 4*C*	KW	Pre: 347 Post: 311 Back: 340	1.0	3.3e-78
		MW U	See previous	Pre vs post: 1.0 Pre vs back: 1.0 Post vs back: 1.0	Pre vs post: 4.2e-08 Pre vs back: 1.8e-81 Post vs back: 4.9e-29
c	Fig. 4*E*, left	KW	See previous	1.0	2.5e-34
		MW U	See previous	Pre vs post: 0.75 Pre vs back: 1.0 Post vs back: 1.0	Pre vs post: 8.9e-04 Pre vs back: 2.1e-38 Post vs back: 3.3e-11
d	Fig. 4*E*, right	KW	See previous	1.0	1.6e-40
		MW U	See previous	Pre vs post: 1.0 Pre vs back: 1.0 Post vs back: 1.0	Pre vs post: 7.1e-08 Pre vs back: 1.2e-44 Post vs back: 3.3e-10
e	Text	WSR	340	1.0	6.1e-43
f	Fig. 5*C*	*t*-test	18	0.57	7.8e-03
g	Fig. 5*F*	*t*-test	18	0.68	3.1e-03
h	Fig. 6*B*	*t*-test	17	0.98	2.8e-05
i	Fig. 6*E*	*t*-test	17	0.54	1.0e-02
j	Extended Data Fig. 6-1*B*	χ^2^	2631	1.0	1

Lower case letters in first column are used as superscripts in text and figure captions. KW, Kruskal–Wallis test; MW U, Mann–Whitney *U* test; WSR, Wilcoxon signed-rank test; *t* test, one-sampled *t* test against zero-mean distribution; *p* values in rows b–d were corrected using the Bonferroni method.

10.1523/ENEURO.0299-18.2019.f6-1 Extended Data Figure 6-1 Survival and positioning of EA-free episodes. ***A***, Survival function for EA-free episodes. EA-free episodes were defined as periods within the background phase that did not contain EA events, i.e., inter-EA event intervals in the background phase. The survival function shows what fraction of EA-free episodes (*y*-axis) lasts for at least a certain time (*x*-axis). Line color indicates animal, data pooled from all animals are shown in black. The average survival function decays logarithmically up to a duration of about 20 s and then exhibits a heavy tail. ***B***, Relative placement of long EA-free episodes (≥25 s, red line in ***A***) within background phases. Long episodes occur throughout background phases with uniform probability (all animals: *N* = 2631, *p* = 1, χ^2^ test^j^). This indicates, that an impending change to high-load dynamics cannot readily be predicted by the presence or absence of long EA-free episodes. Download Figure 6-1, TIF file.

10.1523/ENEURO.0299-18.2019.f6-2 Extended Data Figure 6-2 Surrogate and permutation tests for correlations in Figure 6. ***A***, Significance of the anti-correlation between background rate of low-load bursts and rate of high-load bursts across sessions (left panel; repeated from Fig. 6*A*). We generated surrogate background phases from time series of low-load bursts with durations drawn from the overall distribution of low-load bursts in all background phases, separated by interburst intervals drawn from a Poissonian distribution. The rate parameter of the Poissonian process was set to yield average rate of low-load bursts across all background phases. Significance was assessed by comparing Kendall’s tau of the original data (τ = –0.43, red triangle in right panel) to a distribution of Kendall’s taus obtained from 1000 surrogate correlations. *p*_surrogate_ was defined as the fraction of surrogate correlations below the original correlation. The anti-correlation between background rate of low-load bursts and rate of high-load bursts was significant across sessions (*p*_surrogate_ < 0.001). ***B***, Correlation between background rate of low-load bursts and rate of high-load bursts averaged per animal (left; repeated from Fig. 6*C*). Using a permutation test, we assessed whether animals with on average higher background rates of low-load bursts had significantly higher or significantly lower average rates of high-load bursts. We generated surrogate data by randomly allocating sessions from the overall pool such that each animal was assigned as many sessions as it originally contributed to the dataset. Significance was assessed by comparing the original correlation (τ = –0.33) to 1000 correlations of surrogate animals. The grand average background rate of low-load bursts in an animal did not indicate its grand average rate of high-load bursts (left-tailed *p*_surrogate_ = 0.31). ***C***, Significance of the correlation between the background rate of low-load bursts and the percentage of time spent without EA (left panel; repeated from Fig. 6*D*). Surrogate background phases were generated as described in ***A***. Significance was assessed by comparing Kendall’s tau of the original data (τ = 0.39, red triangle in right panel) to a distribution of Kendall’s taus obtained from 1000 surrogate correlations. *p*_surrogate_ was defined as the fraction of surrogate correlations exceeding the original correlation. The correlation between the background rate of low-load bursts and the percentage of time spent without EA was significant (*p*_surrogate_ < 0.001). ***D***, Permutation test for the correlation between the average background rate of low-load bursts and total percentage of time spent without EA across animals (left; repeated from Fig. 6*F*). Significance was assessed by comparing the original correlation (τ = 0.29) to 1000 correlations from surrogate animals. The grand average background rate of low-load bursts in an animal did not indicate the total percentage of time it spent without EA (*p*_surrogate_ = 0.32). Download Figure 6-2, TIF file.

10.1523/ENEURO.0299-18.2019.f6-3 Extended Data Figure 6-3 Simulation of rate-modulated spike trains. To test the hypothesis that our findings could result from a rate-modulated spiking process, we simulated a surrogate dataset (SDS) of 105 sessions, each lasting 2.5 h, as nested modulated Poisson processes (PPs). Panels ***A–F*** illustrate this procedure for a 35-min segment from a surrogate session. The gain of the rate modulation was varied to simulate the range of high-load burst rates across our recording sessions. ***A***, A first PP (PP_0_) was used to generate a baseline time series of spikes at a fixed rate (0.17 spikes/s). ***B***, To generate bursts at realistic rates, we copied spikes from a second PP (PP_1_; fixed rate 0.28 spikes/s) with a probability given by the modulation function Modulation_1_ to create train_1_. Peaks in Modulation_1_ were timed randomly with a separate PP (dots in ***B***) according to the mean rate of bursts across all sessions (0.92 bursts/min). This time series was convolved with a triangular kernel to create a probability density function for copying spikes. ***C***, Bursts in train_1_ were detected as in the original recordings (spikes closer than 2.5 s were grouped into the same burst; Fig. 1*D*). To assign bursts to low-load and high-load classes, we could not use our original classifier, because the within-burst structure was not replicated. We therefore used the area under the curve (AUC) of the rate profile (black) of each burst to approximate spike load (compare Extended Data Fig. 1-1). From the AUC values we then defined a threshold to separate high-load from low-load burst. The threshold was adjusted to reproduce in SDS_2_ (see ***E***) the 3:1 ratio of the rates of low-load to high-load bursts observed in our recordings, i.e., bursts with AUC values above the 76th percentile were considered high-load bursts, the others were low-load bursts. Note that we neglected medium-load bursts for simplicity. Most but not all bursts in SDS_1_ coincided with peaks in Modulation_1_ with only few qualifying as high-load bursts. ***D***, To reproduce the clustering of high-load bursts we introduced a further slow modulation (Modulation_2_). For simplicity, we used a step function with fixed duration corresponding to the average duration of high-load clusters in the original dataset. Modulation_2_ defined the copying probability of additional spikes from PP_2_ (0.39 spikes/s) into train_1_, resulting in train_2_. Phases with high copying probability were distributed randomly (2.3/h; average high-load cluster rate in the original data). ***E***, Analogous to the procedure in ***C***, we identified low-load and high-load bursts in train_2_, resulting in SDS_2_. ***F***, High-load clusters were defined as in the original data (high-load bursts closer than 3 min were assigned to the same cluster; Fig. 3*D*). For simplicity, we ignored transition phases and thus all remaining periods were considered background phases. ***G***, To simulate different spike rates, we varied the gain (range: 0.1–1.125) of Modulation_2_ and generated 105 surrogate sessions (top: example probability functions with different gains; gain = 1 was used for the examples in ***A–F***). The threshold to distinguish high-load and low-load bursts (compare ***C***, ***E***) was based on the surrogate session generated with gain = 1 and kept constant throughout. The rates of high-load bursts (left) and low-load bursts (right) varied with the gain of Modulation_2_. Magenta dots, Data for gain = 1, corresponding to the example in ***E***, ***F***. ***H***, In SDS_2_, the rate of high-load bursts was positively correlated to that of low-load bursts in background phases (τ = 0.32, *p*_τ_ = 1.2 × 10^−6^). In contrast, these rates were anti-correlated in the original data (inset reproduced from Fig. 6*A*). ***I***, Furthermore, in SDS_2_ the rate of low-load bursts in background phases was significantly anti-correlated to the duration of background phases (τ = –0.14, *p*_τ_ = 1.3 × 10^−3^; *N* = 240, samples were randomly drawn to match the sample size of the original data). In contrast, these rates were positively correlated in the original data (inset reproduced from Fig. 5*E*). Note that the exact τ and *p*_τ_ values for the correlations reported in ***H***, ***I*** varied statistically across different runs of the simulation with the same parameter settings. Across different runs, the general relationships (significant positive correlation in ***H***, significant anti-correlation or no correlation in ***I***) were preserved and never resembled the correlations in the original data. Download Figure 6-3, TIF file.

### Surrogate tests

Significance of burstiness scores (Figs. 1*C*, 3*B*,*C*, 4*D*) and of the correlations shown in Figures 5, 6 was assessed using surrogate tests. Surrogate event series were generated by concatenating intervals derived from Poisson processes. The rate parameter *rate_adjusted_* of the Poisson process was set to yield surrogate series matching the event rates in the original data (*rate_observed_*) after adjustment for dead time (*T_dead_*) and event duration (*T_event_*) as $rate_{adjusted}=\frac{rate_{observed}}{1-rate_{observed}\cdot \left(T_{dead}+T_{event}\right)}$, with *T_dead_* = 0.0833s and *T_event_* = 0 s for surrogate spike series. For surrogate burst series we used *T_dead_* = 3.5 s and defined *T_event_* as the mean duration of burst events occurring during all episodes for which the surrogate was generated. *rate_observed_* was a constant calculated as a duration-weighted average of event rates from all episodes considered, e.g., when assessing burstiness of spikes in all background phases *rate_observed_* was defined as the average rate of spikes across all background phases, weighted by background phase duration. The duration of surrogate bursts was randomly drawn from the pool of burst events in the respective episodes. Surrogate tests based on event series are shown and further described in Extended Data Figures 5-2*A–C*, 6–2*A–C*.

Significance of correlations across the set of animals was assessed with a permutation test. To generate surrogate animals, we randomly permuted episodes, i.e., background phases (Extended Data Fig. 5-2*B*,*D*) or sessions (Extended Data Fig. 6-2*B*,*D*), from the pool of all episodes such that each surrogate animal was randomly assigned as many episodes as it originally contributed to the dataset.

To test significance, the original values were compared to distributions of 1000 surrogate values. The *p* value *p*_surrogate_ was defined as the fraction of surrogate values above (right-tailed) or below (left-tailed) the original value.

## Results

This study addresses the question what patterns of EA occur in LFPs recorded from the hippocampus of kainate-injected mice and how these patterns interact.

### Epileptiform spikes come in bursts

As is typical for this animal model, epileptiform spikes were frequent in our recordings. These spikes occurred mainly as components of larger bursts or aggregated loosely into smaller bursts that contained fewer spikes. In contrast, large, putative behavioral seizures were rare (estimated range: 0.0–9.6/d).

To describe burst patterns systematically, we first detected spikes using a custom algorithm and transformed LFPs into spike trains (Fig. 1*A*,*B*). Spike trains in all sessions had burstiness scores (0.37–0.70) significantly higher than Poissonian surrogate trains (burstiness –0.04 to 0.00; *p* < 0.001 for all recordings; Fig. 1*C*) and non-normal interspike interval distributions (Fig. 1*D*). This corroborated our observation that spikes preferentially occurred in bursts. We then delimited bursts by grouping spikes (Fig. 1*D*), thereby creating time series of two general types of EA events: Bursts and solitary spikes (Fig. 1*E*). Such a time series approach captures the discrete nature of the process more directly and requires fewer parameters compared to an analysis of continuous rate profiles (Extended Data Fig. 1-1).

### Categories of bursts

We observed that bursts did not simply differ in a single property but rather in a composite of multiple features. To classify bursts according to features associated with spike load we used a machine learning algorithm (SOM; Fig. 2*A*,*B*) and assigned a spike load index ranging from 0 (loose aggregation of spikes) to 1 (long spike burst with high rate) to each burst. A hierarchical clustering analysis (Fig. 2*C*; Extended Data Fig. 2-1) allowed us to distinguish three distinct burst categories: low-load, medium-load and high-load bursts. Spike load was loosely linked to spike rate and duration in that high-load bursts showed elevated values for both. Short low-load bursts could also have high spike rates (Fig. 2*D*), whereas long low-load bursts tended to have low spike rates. Medium-load bursts covered intermediate ranges of duration and spike rate in highly variable combinations. Bursts corresponding to visually identified behavioral seizures had some of the highest spike rates and longest durations.

Although this classification was based specifically on the spike statistics of the bursts, bursts within a category showed consistent morphologic features (Fig. 2*B*; Extended Data Fig. 2-2). Bursts corresponding to visually identified large behavioral seizures had spike load index = 1 (*N* = 18). During such bursts, we observed highly synchronous activity on the contralateral side and in the entorhinal cortex (Extended Data Fig. 2-2*A*). The remaining bursts with spike load index = 1 (*N* = 125) and the other high-load bursts with spike load index <1 were accompanied by intense EA in the contralateral dentate gyrus but only sparse spiking in the entorhinal cortex (Extended Data Fig. 2-2*B*,*C*). In low-load bursts, the spike component was typically followed by a clearly discernible wave of opposite polarity, smaller amplitude, and longer duration, similar to a spike-wave discharge (comparable to type 1 spikes in Chauvière et al., 2012). During low-load bursts, contralateral and entorhinal activity was sparse or absent (Extended Data Fig. 2-1*D*). Medium-load bursts often contained periods in which spikes were more densely packed than in low-load bursts and where the wave component was less salient or absent. During medium-load bursts, contralateral activity was typically more pronounced than during low-load bursts.

### High-load bursts form clusters

The temporal succession of the three burst types suggested patterns of higher order: Periods dominated by high-load bursts appeared to alternate with extended phases containing only low-load bursts (Fig. 3*A*). In line with this observation, we found that high-load bursts clustered significantly across all sessions pooled (*p* < 0.001 burstiness; Fig. 3*B*) as well as in most sessions individually (64 sessions significantly clustered with *p* < 0.05, 20 not clustered; Fig. 3*C*). We delimited clusters of high-load bursts as the temporal extent of high-load bursts connected by intervals shorter than 3 min (Fig. 3*D*). Note that within such clusters intervals between high-load bursts were typically not devoid of EA but could contain additional bursts of lower load and solitary spikes (Fig. 3*A*). For consistency, we treated isolated high-load bursts (36%; Fig. 3*E*) as high-load clusters. High-load clusters with more than one high-load burst could last from 20 s up to ∼ 50 min (Fig. 3*F*). The fraction of time spent in high-load clusters fluctuated considerably from session to session (Fig. 3*G*) and was independent of the time elapsed since injection of kainate (τ = 0.01, *p*_τ_ = 0.8) and of the daytime of the recording (ANOVA^a^ contrasting the morning and afternoon group).

### Transitions are rich in low-load and medium-load bursts

High-load clusters were often preceded and followed by aggregations of low-load and medium-load bursts (Fig. 4*A*). We identified these pre-phases and post-phases through change points in the interburst interval series (Extended Data Fig. 4-1*A*). Depressions following high-load clusters were also considered as post-phases and could be concluded by rebound bursts (Extended Data Figs. 4-1*B*, 4–2). Of all high-load clusters, 86% were preceded by pre-phases and 81% followed by post-phases.

After delimiting high-load clusters and transition phases, we defined background phases by exclusion (Extended Data Fig. 4-1*A*). Median spike rate was more than twice as high in the pre-phase and post-phase than in the background phase (Fig. 4*C*).^b^ Spikes in each of the phase types had a strong tendency to occur in bursts (Fig. 4*D*), corroborating that even during background phases spiking was not random but structured in bursts.

Median burst rate was significantly higher in the pre-phase than in the post-phase (Fig. 4*E*).^c,d^ During background phases, low-load bursts occurred at substantially higher rates than medium-load bursts (median rate and range for low-load bursts: 0.84/min, 0.00–1.90/min; for medium-load bursts: 0.06/min, 0.00–0.53/min).^e^ Transition phases thus appeared to be more specifically associated with medium-load bursts than with low-load bursts.

### Background phase duration is positively correlated with burstiness and the rate of low-load bursts

Duration and EA content of background phases varied (Fig. 5*A*, Extended Data Fig. 5-1). We therefore asked, whether there was a connection between the duration of background phases and the rate and structure of EA within them. Across the pool of background phases, the burstiness of spikes within a background phase correlated positively with its duration (τ = 0.27, *p*_surrogate_ = 0.009; Fig. 5*B*; Extended Data Fig. 5-2*A*), i.e., burstier background phases lasted longer. To test whether this relationship held for each animal individually, we calculated least-squares regressions between burstiness and duration across all background phases per animal (Fig. 5*C*). The distribution of regression slopes significantly differed from random (*t*-test^f^, *p* = 7.8 × 10^−3^) and most slopes were positive (15 out of 18 mice), demonstrating a consistently positive relationship between burstiness and duration of background phases. However, animals with on average longer background phases did not score significantly higher overall burstiness (τ = 0.07, *p*_surrogate_ = 0.3; Fig. 5*D*; Extended Data Fig. 5-2*B*). Hence, while overall burstiness of background spiking in a mouse did not indicate the average duration of its background phases, burstiness and background phase duration were positively correlated across background phases of most mice individually.

Background phases also lasted longer if they had higher rates of low-load bursts (τ = 0.28, *p*_surrogate_ < 0.001; Fig. 5*E*; Extended Data Fig. 5-2*C*). This relationship was reproduced for most animals individually (15 out of 18; Fig. 5*F*).^g^ However, the average background rate of low-load bursts of a mouse did not indicate the average duration of its background phases (τ = 0.20, *p*_surrogate_ = 0.5; Fig. 5*G*; Extended Data Fig. 5-2*D*).

### Sessions with higher rates of low-load bursts in background phases have lower rates of high-load bursts

Longer background phases in a recording session would leave less time available for high-load bursts. Alternatively, the fact that long background phases were associated with increased background rates of low-load bursts could be due to higher excitability, which in turn might lead to higher rates of high-load bursts. The former situation would lead to an anti-correlation between the background rates of low-load bursts and the rates of high-load bursts across sessions, whereas the latter would result in a positive correlation. In addition, the clustering of high-load bursts makes it difficult to predict the specific relation between these EA patterns. We found that across sessions, the average background rate of low-load bursts was significantly anti-correlated to the rate of high-load bursts (τ = –0.43, *p*_surrogate_ < 0.001; Fig. 6*A*; Extended Data Fig. 6-2*A*). Regressions across sessions had negative slopes in 15 out of 17 mice (*t*-test^h^ against random distribution of slopes: *p* = 2.8 × 10^−5^; Fig. 6*B*). This indicates that the inverse relationship between the background rate of low-load bursts and the rate of high-load bursts is robust across mice. Again, there was no significant (anti-)correlation between the average rate of high-load bursts and the average background rate of low-load bursts of a mouse (τ = –0.33, *p*_surrogate_ = 0.05; Fig. 6*C*; Extended Data Fig. 6-2*B*). The background rate of low-load bursts thus more closely reflected the state of a mouse during a session than its overall condition.

Although elevated background rates of low-load bursts were linked to fewer high-load bursts in a session, low-load bursts themselves are also pathologic activity. We therefore tested whether the increased rates of low-load bursts in longer background phases would increase the overall time spent with EA. Across sessions, the percentage of time spent without EA in background phases was larger in sessions with higher background rates of low-load bursts (τ = 0.39, *p*_surrogate_ < 0.001; Fig. 6*D*; per animal: Fig. 6*E*^i^; across animals: Fig. 6*F*; survival probability of EA-free episodes: Extended Data Fig. 6-1*A*). In addition, the probability for long EA-free episodes did not decrease with increasing background phase duration (Extended Data Fig. 6-1*B*). We thus rejected the hypothesis that elevated background rates of low-load bursts curtail the overall time spent without EA.

In summary, the background rate of low-load bursts was significantly correlated to (1) the duration of the background phase and (2) to the percentage of time spent without EA, but was (3) significantly anti-correlated to rate of high-load bursts. We found that the background rate of low-load bursts more strongly correlated with these signatures of susceptibility than the background rate of spikes or solitary spikes and that these results were robust against changes in event definition (Extended Data Fig. 5-3).

## Discussion

We analyzed the temporal structure of epileptiform spiking in LFPs recorded in a mouse model of MTLE. Since large parts of EA consisted of well-defined spike bursts, we chose an approach based on time series. Time series are well suited for correlation analyses to investigate statistical interactions between event types.

In previous studies, large EA events have been described by features such as duration, spike rate, spike amplitude, spike wave form, and the evolution of these parameters over time (Twele et al., 2016). To avoid effects resulting from variations in recording conditions across animals and time, such as electrode properties, glial scarring, etc., we classified bursts based on a set of features best summarized as spike load. Importantly, spike load was only loosely correlated to burst duration and spike rate within a burst; it could differ considerably for bursts of the same duration or spike rate.

Although they were classified according to statistical measures of their spike structure, events in a burst category were morphologically similar. Low-load bursts typically consisted of spikes followed by longer deflections of opposite polarity with lower amplitudes. These bursts were similar to trains or groups of spike-wave discharges reported previously for this animal model (Riban et al., 2002; Twele et al., 2016). Medium-load bursts typically comprised spike trains with higher rates, where wave components were less apparent or obfuscated by subsequent spikes.

In high-load bursts, most spikes were densely packed. High-load bursts often started with loosely spaced spike-wave discharges and evolved into dense spiking with progressively lower amplitudes, like type A bursts described by Chauvière et al. (2012) for kainate injected rats. In terms of both morphology and incidence, high-load bursts were similar to what other studies using our animal model labeled as hyperparoxysmal discharges, high voltage sharp waves, or electrographic seizures based on visual inspection and manual classification (Riban et al., 2002; Twele et al., 2016; Zeidler et al., 2018). With respect to long-term dynamics, the clustering of high-load bursts appeared similar to seizure clusters commonly described in rodent models (Lim et al., 2018) and in patients (Karoly et al., 2017). A small fraction of high-load bursts were similar to large behavioral seizures described elsewhere based on their characteristic, stereotypical wave form and highly synchronous activity across recording sites (Riban et al., 2002; Zeidler et al., 2018). Their incidence was comparable to other reports on various rodent models of epilepsy (Rattka et al., 2013; Klee et al., 2017). We did not investigate these in further detail because they were too rare for a meaningful correlation analysis.

Differences between burst categories also manifested in their temporal occurrence. Clusters of high-load bursts were often preceded by aggregations of low-load and medium-load bursts and alternated with phases populated almost exclusively by low-load bursts. This temporal cohesion of morphologically similar events substantiates our classifications and suggests distinct temporal dynamics dominated by different types of bursts. Segregating such temporal phases of distinct EA dynamics is crucial to evaluate relationships between different types of EA. Separating transition phases rich in low-load and medium-load bursts enabled us to specifically address EA in background phases and to investigate how background activity relates to the rate of high-load bursts.

Our findings indicate that the interpretation of low-load bursts should depend on temporal and functional context: While the rates of low-load and medium-load bursts increased before the onset of high-load dynamics, background rates of low-load bursts were anti-correlated to rates of high-load bursts. We further found that higher rates of low-load bursts during background phases were associated with an increased duration of the background phase and a larger relative amount of time spent without any EA. This antagonistic relationship between low-load bursts and susceptibility to high-load bursts is in agreement with the hypothesis that epileptiform spikes could reduce seizure susceptibility (Barbarosie and Avoli, 1997; De Curtis and Avanzini, 2001; Khosravani et al., 2003; Muldoon et al., 2015; Goncharova et al., 2016). On a mechanistic level, epileptiform spikes, and especially the wave component following the sharp spike during spike-wave discharges, have been proposed to reflect GABAergic inhibitory processes (De Curtis and Avanzini, 2001; Muldoon et al., 2015). In our recordings, low-load bursts mostly consisted of spike-wave discharges and thus might be interpreted as a signature of boosted inhibition. Conversely, in medium-load bursts, the wave was often less prominent and spikes were more densely packed. While this could be due to an overlap on the signal level, the increase of medium-load bursts preceding high-load clusters could also indicate a gradual breakdown of inhibition. Along the same line, high rates of low-load and medium-load bursts during transition phases could promote high-load dynamics through excessive GABAergic input: high rates of spikes might increase the intracellular chloride concentration and hence make GABA act depolarizing (Pallud et al., 2014; Magloire et al., 2019), thereby enhancing excitability and facilitating high-load bursts. Alternatively, an increase of intracellular chloride concentration could trigger potassium extrusion into the extracellular space and thus enhance excitability (De Curtis and Avoli, 2016; Librizzi et al., 2017).

One could argue that the existence of different categories of bursts with lower or higher spike load and the alternation we observe between background, transition phases and high-load clusters is governed by modulations of the spike rate. Such modulations and an accompanying buildup in susceptibility have been suggested to be due to slowly changing variables such as extracellular ion concentrations or metabolic factors. For example, a slow “permittivity variable” has been used in computational models of seizure generation (Jirsa et al., 2014). While it is possible that underlying slow modulations contributed to transitions from background to high-load dynamics observed in our data, such modulations alone cannot explain the antagonistic relationship between the rate of low-load bursts in background phases and the susceptibility to high-load bursts. When simulated as rate-modulated Poisson processes (Extended Data Fig. 6-3), nested modulations indeed reproduced high-load bursts and clusters of high-load bursts (Extended Data Fig. 6-3*A–F*) but they did not result in an anti-correlation between the rate of high-load bursts and the background rate of low-load bursts found in our data (compare Fig. 6*A*, Extended Data Fig. 6-3*H*). Neither did this spike rate modulation replicate the observed positive correlation between the rate of low-load bursts in the background phase and the duration of background phases (compare Fig. 5*E*, Extended Data Fig. 6-3*I*). To reproduce the correlation statistics we observed would require an additional interaction between the rate of low-load bursts in background phases and high-load dynamics.

The ideas of a slow process modulating excitability and a context-dependent interaction between high-load dynamics and background bursting, however, may be reconciled. Based on experiments and computational modeling, Chang et al. (2018) recently proposed that epileptiform spikes interact with slowly increasing network excitability in a context-dependent manner: When the excitability of the network was low, epileptiform spikes further reduced excitability and thus lengthened interictal states. In a transition range of excitability, sufficiently strong, synchronized firing of a population of neurons could prematurely trigger seizures. In analogy to Chang et al. (2018), high rates of low-load bursts could stabilize background phases in our animals, counteracting a continuous increase of network excitability. Low rates of low-load bursts, however, would allow a net increase of excitability. In the transition range of excitability, increased rates of low-load events could then eventually facilitate high-load dynamics. Since bursts of spikes were a more powerful correlate to high-load susceptibility, we hypothesize that spikes grouped into bursts might be particularly effective in keeping excitability in check and thus prolong background dynamics.

In summary, we characterized the nested structure of EA patterns in the kainate mouse model of MTLE using time series analyses and machine learning techniques. Systematically segregating periods of intense high-load bursting and transition phases from background phases allowed us to address the relationship between different types of EA specific to temporal context. Our study corroborates the hypothesis that the role of low-level EA depends on the current state of network dynamics and suggests that the rate of bursts with low spike load could be a powerful correlate of susceptibility dynamics.

## Synthesis

Reviewing Editor: William Stacey, University of Michigan Decisions are customarily a result of the Reviewing Editor and the peer reviewers coming together and discussing their recommendations until a consensus is reached. When revisions are invited, a fact-based synthesis statement explaining their decision and outlining what is needed to prepare a revision will be listed below. The following reviewer(s) agreed to reveal their identity: NONE.

Dr. Bernard and I have discussed this resubmission. You have made considerable improvements to the paper and in general it is of interest for publication. There are however some necessary textual corrections that would make it more clear prior to acceptance.

1. The additional supplemental data are important but often quite complex. The methods section was OK. But it would be better to reduce the explanations in the “results” section to very brief statements that do not interrupt the flow of the rest of the paper. I.e. “permutation tests showed that the p value was XXX (see Fig. 5-1)”. This is especially true when talking about the extended data in 5-2 and 6-2, which are very abstract, and do not really help with the narrative. In fact, it is hard to tell what the data are even showing, but it seems this is just validation of specific parameters. That does not need major effort in the main paper. It would be better to describe them very generally (and briefly) in the paper, and leave the larger details to the figure itself.

2. Regarding the permutation tests, please clarify what it means when the individual surrogate tests (5-1 and 6-1 a,c) were significant but the population permutation tests (5-1 and 6-1 b,d) were not. That section was confusing.

3. There are panels in many of the figures that are not referred to in the paper. Either remove them or describe the results in the text

4. After making those changes, please assure the paper maintains a “bird's eye” view in which it is clear why each test is being done and what it shows. At present it is difficult to follow the paper with all the issues described in #1-3

5. The final conclusions in the discussion assume a causal link between low level bursts and the risk of high level bursts. Those ideas are OK for discussion, but causality was not proven by these data. Thus the statements in the abstract and significance statements that claim a “stability” effect that low level activity “promotes” and that low level EA “can reduce the excitability of the network” should be changed. Your results are consistent with that hypothesis, but there are other possibilities, and causality is not proven. Please add some qualifications to the final statements in the abstract and significance.

6. The thought experiment in lines 617-646 (“while the exact outcome of this consideration depends...”) does not convince and it is difficult to follow. It would be a better idea to contrast your hypothesis with some others with some brief explanation, rather than trying to disprove them by deductive reasoning.

7. Couldn't these data be produced by a slow process that increases epileptogenicity that waxes and wanes? This idea was brought up in a previous review and the authors stated it was beyond the scope of the research. However now with the surrogate data it may not be. This would correspond to a change in the Poisson parameter over shorter epochs in the model of spikiness. This seems quite capable of producing both high-load bursting and longer intervals of it. It was not clear how this parameter was chosen in the tests-was it an average value, a running value, or something else? Please clarify whether the Poisson value was a single value for the entire test, or if it could vary. And comment on how your data may or may not correlate with the idea of a changing poisson variable. This idea seems in line with the final statement “low level EA depends on the current state of network dynamics and suggests the rate of bursts...could be a powerful correlate of susceptibility dynamics”, so it is surprising this idea was not clearly stated.

## Author Response

Dear Dr. Stacey, thank you very much for the review. We're glad that we could clarify the misunderstandings with respect to our analyses. We have changed the manuscript as follows to address the specific points in your latest review.

Overview of changes in figures/extended data

Main figures

• Fig. 4: 4A-D deleted (see P3 below for further details); numbering of subsequent panels changed accordingly; 4J removed

• Fig. 5: 5B and D now in extended data Fig. 5-1; previous 5C now shown as inset in new 5C; numbering of subsequent panels changed accordingly

Extended data

• new Fig. 5-1: previously in Fig. 5BD

• updated index Fig. 5-2 (surrogate/permutation statistics): previous Fig. 5-1

• updated index Fig. 5-3 (robustness against threshold changes, marker comparison): previous Fig. 5-2

• swapped index Fig. 6-1 (survival statistics and positioning of EA-free episodes): previous Fig. 6-2; swapped index to preserve logical order

• swapped index Fig. 6-2 (surrogate/permutation statistics): previous Fig. 6-1; swapped index to preserve logical order

• new Fig. 6-3 (simulation of rate-modulated processes): added to address rate modulated Poisson processes (see P6 in replies)

In the following, text in italics is quoted from your email. Line (L-) and page (p-) numbers refer to the merged PDF file. Note that there is a slight mismatch between the line numbers in this file and the docx version.

P1: Redundancy between Methods/Results; too much diversion from main narrative

1. The additional supplemental data are important but often quite complex. The methods section was OK. But it would be better to reduce the explanations in the “results” section to very brief statements that do not interrupt the flow of the rest of the paper. I.e. “permutation tests showed that the p value was XXX (see Fig. 5-1)”. This is especially true when talking about the extended data in 5-2 [now 5-3] and 6-2 [now 6-1], which are very abstract, and do not really help with the narrative. In fact, it is hard to tell what the data are even showing, but it seems this is just validation of specific parameters. That does not need major effort in the main paper. It would be better to describe them very generally (and briefly) in the paper, and leave the larger details to the figure itself.

A1: We thank the reviewers for pointing out the methodological overload in our results section. We now streamlined the results section and better explain the objectives of the extended data in their respective captions. Details are now restricted to the methods section (definition of bursts; categorization of bursts; definition of high-load clusters) and we only briefly mention the most important analysis steps in the results where necessary. Regarding the presentation of data from the previous Fig. 5-2 and 6-2 we made the following changes:

• Fig. 5-2 (now Fig. 5-3, showing robustness of burst delimitation threshold and marker comparison): We now explain why this analysis was undertaken directly in the caption and only briefly mention the essential outcome in the results (L-437-440).

• Fig. 6-2 (now Fig. 6-1, showing survival and positioning of EA-free episodes): This data set shows that longer EA-free episodes are evenly distributed throughout the background phase and thus cannot be used as indicators of upcoming high-load dynamics in our data. We now explain the purpose of this analysis directly in the caption.

P2: Difference between within animal and across average animal correlation

2. Regarding the permutation tests, please clarify what it means when the individual surrogate tests (5-1 and 6-1 a,c) were significant but the population permutation tests (5-1 and 6-1 b,d) were not [now 5-2 and 6-2]. That section was confusing.

A2: We now explain in the results how to interpret the observation that the correlations between variables were significant across background phases (Figure 5BE), resp. sessions (Figure 6AD), but not when averaged across all background phases (Figure 5DG; L-390-407), resp. sessions of an animal (Figure 6CF; L-417-426). For example, w.r.t. Figure 5B/5-2A we now write in the results (L-399-402): “..., while overall burstiness of background spiking in a mouse did not indicate the average duration of its background phases, burstiness and background phase duration were positively correlated across background phases of most mice individually.”

P3: Unmentioned panels

3. There are panels in many of the figures that are not referred to in the paper. Either remove them or describe the results in the text

A3: We now either mention the respective panels in the text, moved them to the extended data (and mention them in the relevant main figure caption) or removed them altogether:

Previously not addressed in the text but now mentioned:

• Fig. 3EF (duration and size of high-load clusters), now mentioned in L-361-364

• Fig. 5C (N for background phases per animal): combined with new Fig. 5B as an inset

Panels moved to extended data:

• Fig. 5BD (B: fraction of time spent in background phases, D: survival function of background phases) are now presented in Figure 5-1AB. We did not want to remove these panels altogether, because they provide valuable statistics on the variability, prevalence and duration of background phases to the interested reader.

Removed:

• Fig. 4J (fraction of time occupied by medium-load and low-load bursts in pre-phases, post-phases and background phases): Considering that we show the rate of medium-load and low-load bursts in the respective phases in Fig. 4E, we removed panel 4J because it did not add enough new information.

• Please note that we also completely removed Fig. 4A-D, although these panels were mentioned in the previous version of the manuscript, to avoid misinterpretation of our data. In these panels, the median rate of spikes showed a continuous increase of the spike rate before high-load clusters, suggesting a gradual buildup in the high-load cluster triggered averages/medians. We now tested if this was valid for individual transitions by calculating regressions between spike timing relative to high-load cluster onset and instantaneous spike rate for each transition. The distribution of regression slopes, however, was not significantly different from random (Student's t-test), indicating that there was no significant trend towards higher spiking as high-load clusters approached for individual transitions. Instead, the impression of a gradual increase in average median spike rate before high-load clusters was due to a bias introduced by short transition phases that generally had higher spike rates than longer ones. Since short transition phases were much more frequent than long ones (Fig. 4A in the previous version of the ms), they contributed more to the averages immediately before onset, resulting in a seemingly continuously increasing spike rate. We think that readers could misunderstand our data as showing that high-load clusters can be predicted as early as 14 min before onset (see previous Fig. 4D), which we did not find, and therefore removed these accessory panels that did not provide information essential for the narrative.

P4: Flow and “bird's eye view”

4. After making those changes, please assure the paper maintains a “bird's eye” view in which it is clear why each test is being done and what it shows. At present it is difficult to follow the paper with all the issues described in #1-3.

A4: We now focus the results more strongly on the main message and provide details in the extended data where appropriate. All tests are now embedded in statements regarding their purpose and outcome. We also revised the whole ms for general readability and flow (connecting sub-topics, structure, wording). We removed the description of the thought experiment from the discussion (see reply to P6) and instead added a simulation substantiating the respective conclusions in the extended data (Fig. 6-3). This is briefly addressed in the discussion.

P5: Abstract and significance statement

5. The final conclusions in the discussion assume a causal link between low level bursts and the risk of high level bursts. Those ideas are OK for discussion, but causality was not proven by these data. Thus the statements in the abstract and significance statements that claim a “stability” effect that low level activity “promotes” and that low level EA “can reduce the excitability of the network” should be changed. Your results are consistent with that hypothesis, but there are other possibilities, and causality is not proven. Please add some qualifications to the final statements in the abstract and significance.

A5: We never claimed causality. In the previous ms the full sentence in the Significance Statement reads

“Both findings are in agreement with the hypothesis that low-level epileptiform activity can reduce the excitability of the network and that its impact depends on the current state of the network.”

and in the abstract

“Our findings support the hypothesis that low-level epileptiform activity can promote network stability but can also participate in transition dynamics towards high-load epileptiform events, depending on the current state of the network.”.

We have now changed the “support” statement and also changed can to could to emphasize the conjunctive in these sentences. These statements are otherwise correct. We did not invent this hypothesis, do cite the corresponding literature and compare it to other hypotheses in the Introduction (previous ms p-3 L-69-78; now still p-3 L-69-78). We again compare it to other publications in the Discussion, providing references that suggest several mechanisms (previous ms p-23, L-571-601; now p-20 L-490-543). We cannot possibly address all these in the space given for the significance statement or abstract. This we do in the discussion and with the new Figure 6-3 (see also P6). We are convinced, however, that it is helpful for the reader to embed our findings in current research and indicate our interpretation of the results (as detailed in the Discussion) in the abstract and significance statement.

P6: Thought experiments

6. The thought experiment in lines 617-646 (“while the exact outcome of this consideration depends...”) does not convince and it is difficult to follow. It would be a better idea to contrast your hypothesis with some others with some brief explanation, rather than trying to disprove them by deductive reasoning.

A6: We now address the hypothesis of rate modulation more concisely in the text (Discussion L-510-528) and replaced the thought experiment by a simulation of rate modulated Poisson processes (shown in extended data Fig. 6-3). The simulation clearly shows that while such a process can produce clusters of high-load bursts, it does not generate the correlations we describe:

• the positive correlation between the background phase duration and the rate of low-load bursts during these phases (compare Fig 5E to 6-3I).

• the anti-correlation between the rate of high-load bursts and that of low-load bursts during background phases (compare Fig. 6A to 6-3H),

• It does also obviously not produce the positive correlation of the duration of background phases and the degree of burstiness of spiking in that phase (Fig 5BC, 5-2A).

Variations of the model's parameters would lead to comparable outcomes. To reproduce the correlations would require interactions in time between the processes defining the activity and duration of the background phase and those defining high-load bursting (Discussion L-526-528). Such interactions cannot be modeled with independent rate-modulated Poisson processes but might be accounted for in the type of model presented by Chang et al. 2018 as discussed in L-529-543.

P7 seems to consist of several questions:

P7a: Slow modulation of spike rate functions

7. Couldn't these data be produced by a slow process that increases epileptogenicity that waxes and wanes? This would correspond to a change in the Poisson parameter over shorter epochs in the model of spikiness. This seems quite capable of producing both high-load bursting and longer intervals of it.

A7a: Increasing the spiking rate could indeed create high-load bursting and high-load clusters and we explained how this could happen within a session in the previous ms on p-25, L-621-634. We now show this in more detail in Fig. 6-3 and discuss it in the current revision (L-510-528; see A6 above).

We do not know what process modulates high-load burst rates over periods of days (or sessions). Our observation just shows that in some sessions it is higher than in others and that this is systematically correlated with lower rates of low-load bursts in background phases for a given animal. The model in Fig. 6-3 includes varying modulations of the high-load burst rate (via the gain of Modulation2) to mimic the variability across sessions but cannot produce the correlations we find. “Epileptogenicity” seems to be different from general excitability, if increasing spike/burst rates are considered as such. Modulation of the latter would be something like varying the gain of Modulation2.

P7b: Rate parameters in Poisson processes of surrogate data

... It was not clear how this parameter was chosen in the tests-was it an average value, a running value, or something else? Please clarify whether the Poisson value was a single value for the entire test, or if it could vary.

A7b: In all tests based on Poisson processes (tests for burstiness and clustering in Figs. 1C, 3BC, 4D; surrogate correlation tests in Figs. 5-2AC; 6-2AC) the Poisson rate parameter was kept constant. The rate parameter was derived from the duration-weighted average of the respective event rates. We now emphasize that the rate parameter was a constant (Methods, L-290-293):

“rateobserved [i.e. rate of spikes respectively of bursts of a given category] was a constant calculated as a duration-weighted average of event rates from all episodes considered, e.g. when assessing burstiness of spikes in all background phases rateobserved was defined as the average rate of spikes across all background phases, weighted by background phase duration.”

Note that when testing for burstiness of spikes (Fig. 1C and 4D) and clustering of high-load bursts (Fig. 3BC), changing the rate parameter of the Poisson process is not permissible since we need to test against the distributions of intervals a Poisson process would create. Throughout the surrogate tests, the null-hypothesis was that the observed correlation could have been produced by a random (Poissonian) distribution of events. Introducing a fluctuating Poisson parameter would create serial correlations that destroy randomness.

P7c: Discussion of rate modulation

7c: ... And comment on how your data may or may not correlate with the idea of a changing poisson variable.

A7c: We address this in the new ms on p-21, L-519-528:

“When simulated as rate-modulated Poisson processes (Figure 6-3), nested modulations indeed reproduced high-load bursts and clusters of high-load bursts (Figure 6-3A-F) but they did not result in an anti-correlation between the rate of high-load bursts and the background rate of low-load bursts found in our data (compare Figure 6A to Figure 6-3H). Neither did this spike rate modulation replicate the observed positive correlation between the rate of low-load bursts in the background phase and the duration of background phases (compare Figure 5E to Figure 6-3I). To reproduce the correlation statistics we observe would require an additional interaction between the rate of low-load bursts in background phases and high-load dynamics.”

... This idea seems in line with the final statement “low level EA depends on the current state of network dynamics and suggests the rate of bursts...could be a powerful correlate of susceptibility dynamics”, so it is surprising this idea was not clearly stated.

The full sentence reads “Our study corroborates the hypothesis that the role of low-level EA depends on the current state of network dynamics and indicates that the rate of bursts with low spike load is a powerful correlate of susceptibility dynamics.” The way you cite our text suggests that the rate of any type of burst could be used to assess susceptibility. We describe, however, that increased rates of low-load bursts in the background phase correlate with lower rates of high-load bursts in a session, which is counterintuitive if one assumes a slow modulation of burst rates only (see Fig. 6-3). In our view, the modification you suggest would obscure this anti-correlation, which we consider as the main finding in our manuscript.
