# Supplementary figures and images for: Bursts with High and Low Load of Epileptiform Spikes Show Context-Dependent Correlations in Epileptic Mice
Source: eNeuro. 2019 Sep 5;6(5):ENEURO.0299-18.2019. doi: 10.1523/ENEURO.0299-18.2019 (PMC6731539; doi:10.1523/ENEURO.0299-18.2019)

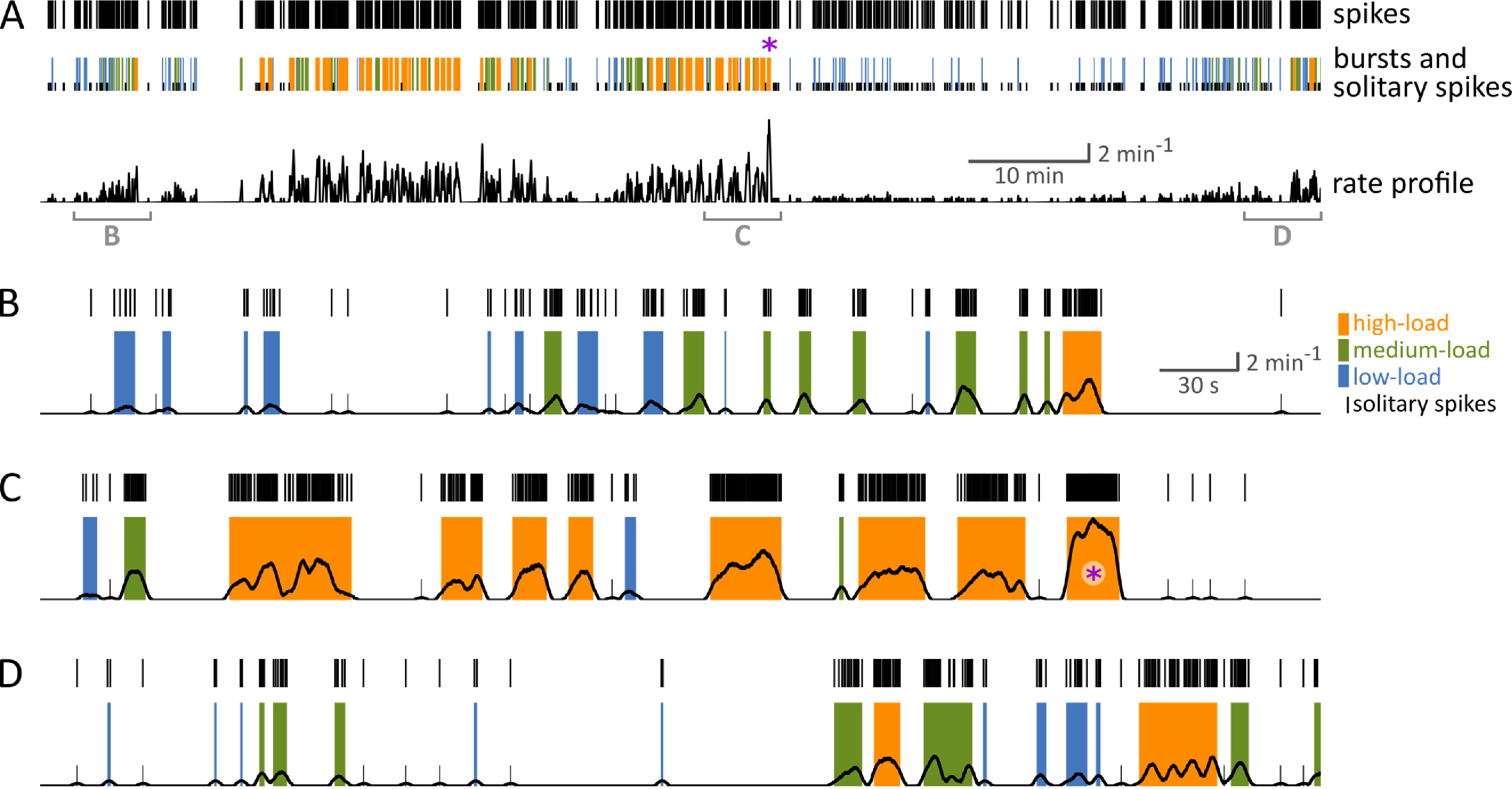

Supplement: Extended Data Figure 1-1 — Comparison between rate profile and detected bursts. A, top, Sequence of epileptiform spikes detected in the LFP shown in Figure 1A. Middle, By delimiting spike bursts (Fig. 1D), we derived time series of solitary spikes (black tick marks) and bursts (colored rectangles). Bursts are colored according to category as detailed in Figure 2. The purple asterisk marks a visually identified, putative behavioral seizure. Bottom, Rate profile generated by convolving the spike times (top) with a Gaussian kernel (width: 5 s, SD: 1.66 s). Generating rate profiles with other kernel shapes and widths yielded similar results. Zooming (B–D) reveals that detected bursts coincide with separable peaks or chains of peaks (e.g., D, last high-load burst) in the rate profile. This corroborates the notion that the bursts detected by our time series approach are indeed distinct events. Extracting such events from a rate profile would have yielded similar results but would have required heuristic tuning of more parameters. Download Extended Data Fogire 1-1, TIF file. [file sup_enu-eN-NWR-0299-18-s03.tif]

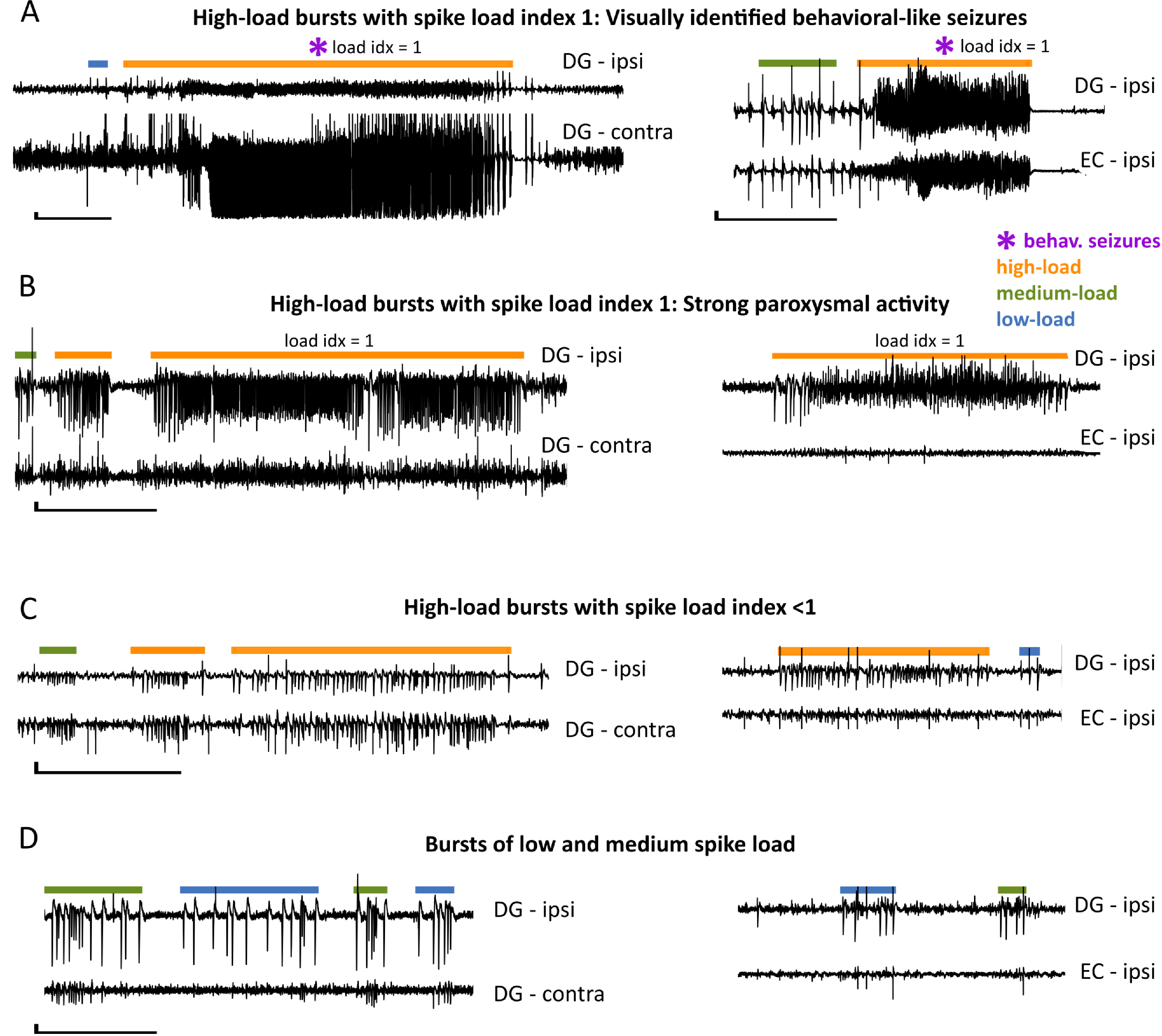

Supplement: Extended Data Figure 2-2. — Representative EA from the kainate-injected dentate gyrus, the contralateral dentate gyrus, and the entorhinal cortex. A, Visually identified, putative behavioral seizures in the ipsilateral dentate gyrus (DG-ipsi, top traces) were accompanied by strong high-frequency and high-amplitude activity on the contralateral side (DG-contra, left), and generalized to the entorhinal cortex (EC-ipsi, right). Horizontal bars delimit automatically detected ipsilateral events and are colored according to the category assigned. Visually identified seizures scored spike load index 1. B, Other bursts with spike load index = 1 were reliably accompanied by intense contralateral EA, but only little synchronous activity in the entorhinal cortex. C, Likewise, ipsilateral high-load bursts with spike load index of <1 often had synchronous EA in the contralateral dentate gyrus with sparser spiking in the entorhinal cortex. D, Medium-load and low-load bursts were morphologically similar to trains of spike-and-wave complexes. During low-load bursts the wave-component was salient, while during medium-load bursts spike patterns typically became denser as spike amplitudes decreased and wave-components appeared less prominent. During medium-load bursts, we typically observed contralateral spiking and sparse spiking could also occur in the entorhinal cortex. Scale bars: 20 s, 1 mV. DG-ipsi/DG-contra pairs (left column) originate from dataset A, DG-ipsi/EC-ipsi pairs (right column) from dataset B. Download Extended Data Figure 2-2, TIF file. [file sup_enu-eN-NWR-0299-18-s05.tif]

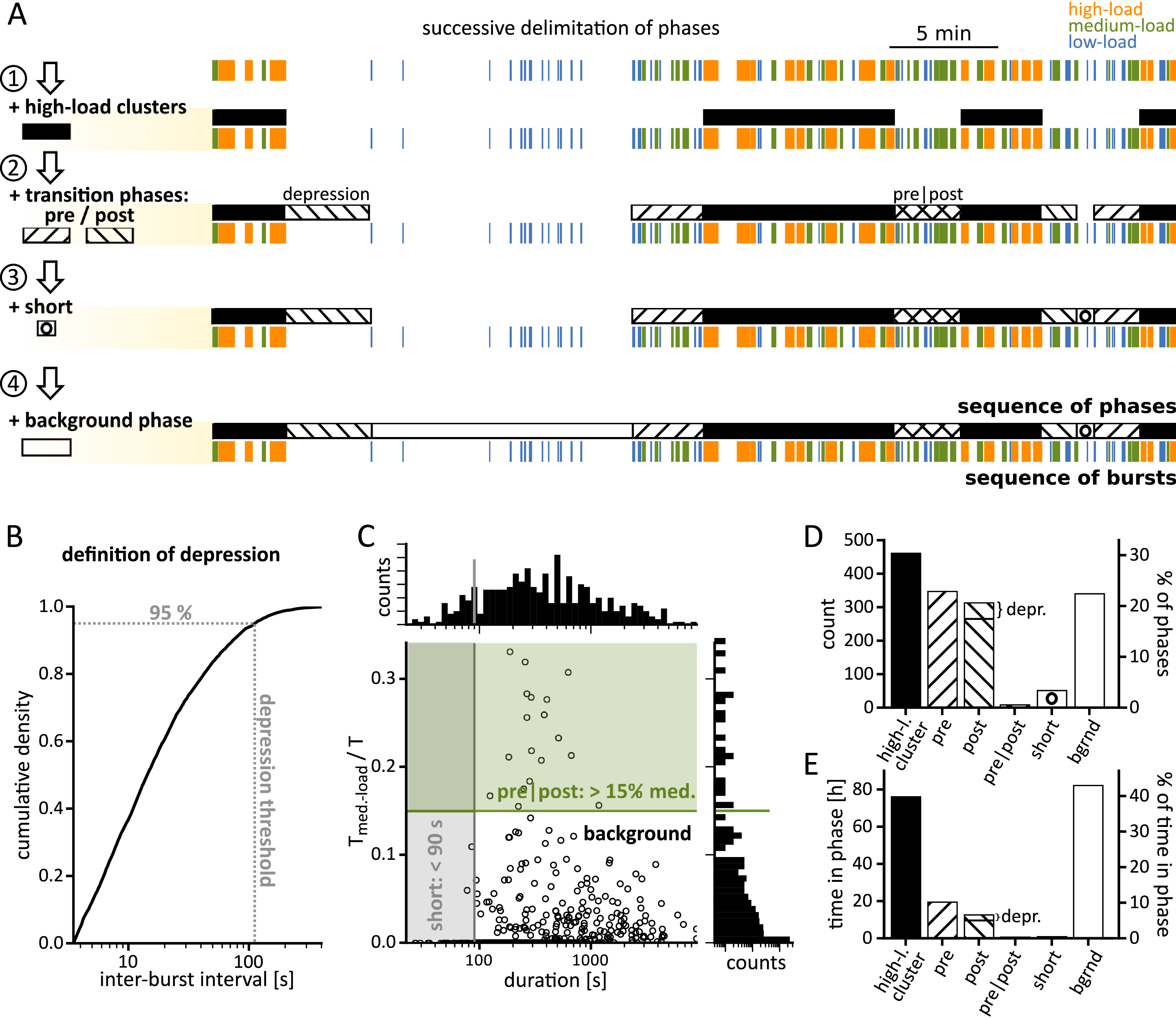

Supplement: Extended Data Figure 4-1 — Step-wise definition of high-load clusters, transition phases and background phases. A, High-load clusters, transition and background phases were derived from the sequence of classified bursts (rectangles, colored according to category). □ High-load bursts closer than 3 min were grouped into the same high-load cluster (black boxes, see Fig. 3). □ Regions rich in medium-load and low-load bursts around high-load clusters were delimited (pre and post, hatched) using change-point analysis. Post-phases could also consist of long burst-free intervals following high-load clusters (“depression,” see B and Extended Data Fig. 4-2A). In a few cases, short lapses between two high-load clusters were occupied by dense clusters of medium-load bursts (“pre|post”). These were identified based on the distribution shown in C. □ + □ From the remaining epochs, we only defined periods longer than 90 s as background phases (C). B, Cumulative interburst interval distribution within periods that neither belonged to high-load clusters nor to pre-phases, nor to post-phases rich in bursts (Nintervals = 6622). A threshold of 112 s (95th percentile of these intervals) was used to identify unusually long intervals following a high-load cluster as depression. C, Fraction of time occupied by medium-load bursts in a period vs. duration of that period shown for periods selected as in B, excluding depression periods. Pre|post indicates periods with more than 15% of time occupied by medium-load bursts. Periods shorter than 90 s were defined as short. D, Occurrence of the different phases in the full dataset. High-load clusters were most abundant, while short and pre|post phases were rare. E, Total time spent in each phase. High-load clusters and background phases each accounted for about 40%. Download Figure 4-1, TIF file. [file sup_enu-eN-NWR-0299-18-s06.tif]

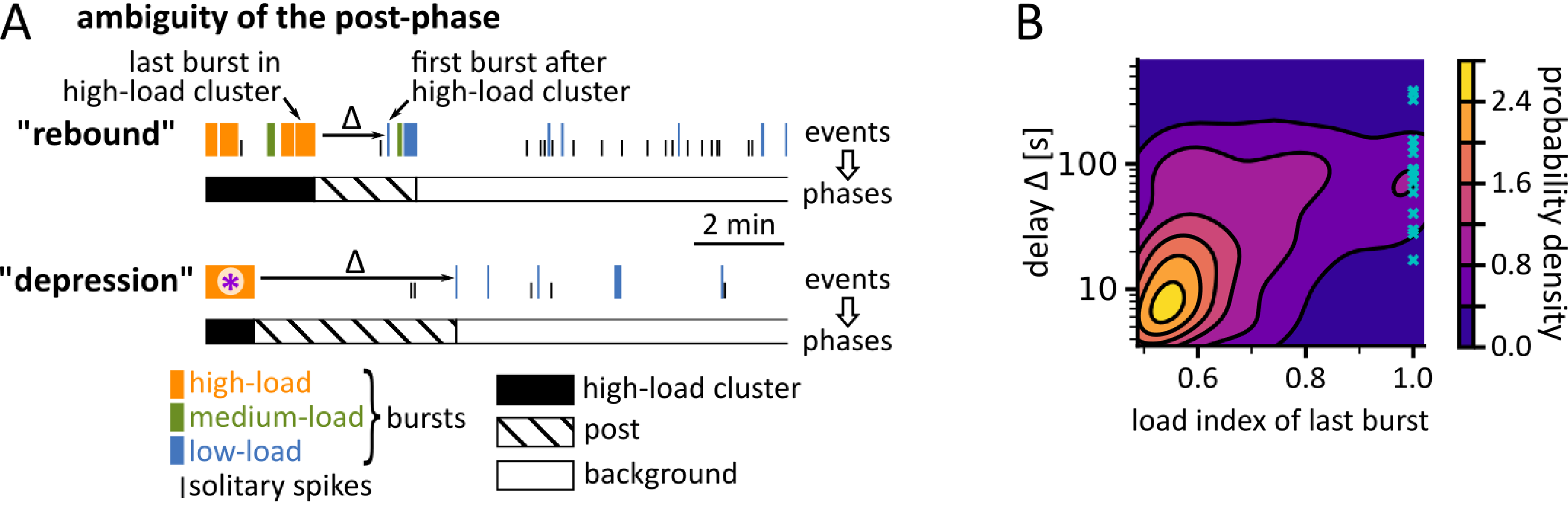

Supplement: Extended Data Figure 4-2 — The dual nature of the post-phase. A, Two examples of periods following high-load clusters. Sequences of EA events are shown above, the phases derived from them are shown below. A high-load cluster could be followed by an episode without bursts terminated by a cluster of bursts (“rebound”). The second example shows a period in which a high-load cluster ended in a visually identified seizure and was followed by a long period without bursts and without obvious rebound (“depression”). Δ denotes the delay between the end of the last burst, which must be a high-load burst by definition, in a high-load cluster and the start of the first following burst. B, Correlation between spike load index of the last burst in a high-load cluster and delay Δ (N = 409, τ = 0.28, pτ = 8.5 × 10^−18^). Blue crosses mark the values obtained for visually identified seizures. High-load clusters ending with bursts of higher spike load are followed by longer delays. Download Figure 4-2, TIF file. [file sup_enu-eN-NWR-0299-18-s07.tif]

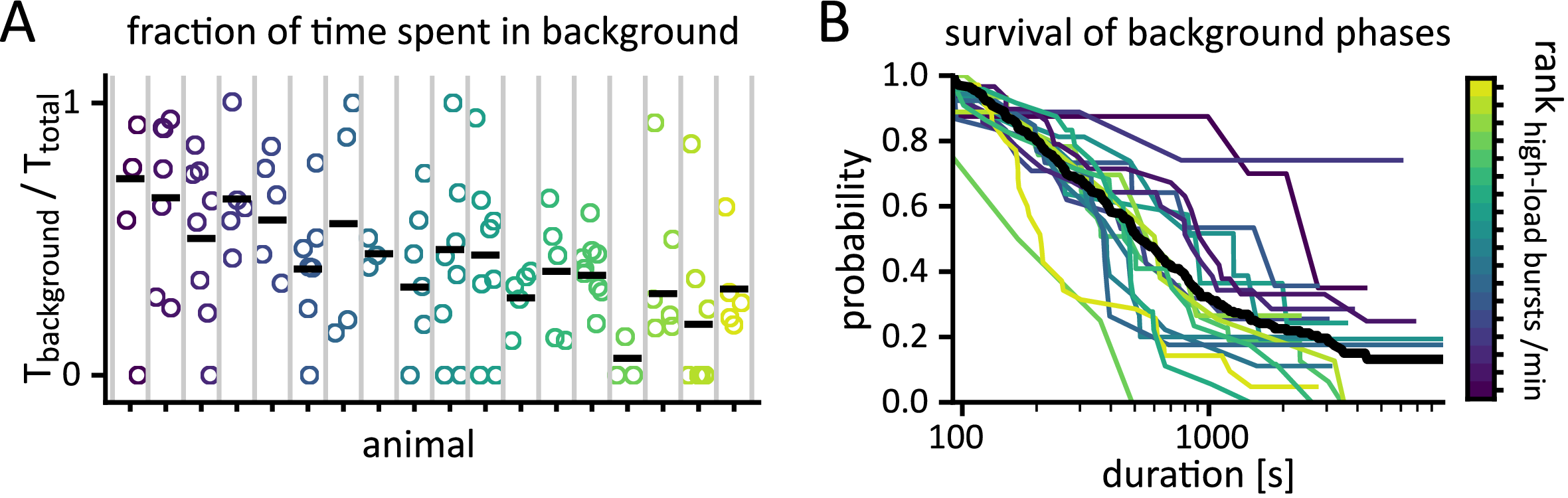

Supplement: Extended Data Figure 5-1 — Fraction of time spent in background phases and survival function. A, Fraction of time spent in background phases for all sessions sorted according to animal. Horizontal dashes indicate medians across recording sessions of the same mouse. B, Survival functions of background phases from all mice individually (colors) and across mice (black). The survival function shows what fraction of background phases (y-axis) lasts for at least a certain time (x-axis). Download Figure 5-1, TIF file. [file sup_enu-eN-NWR-0299-18-s08.tif]

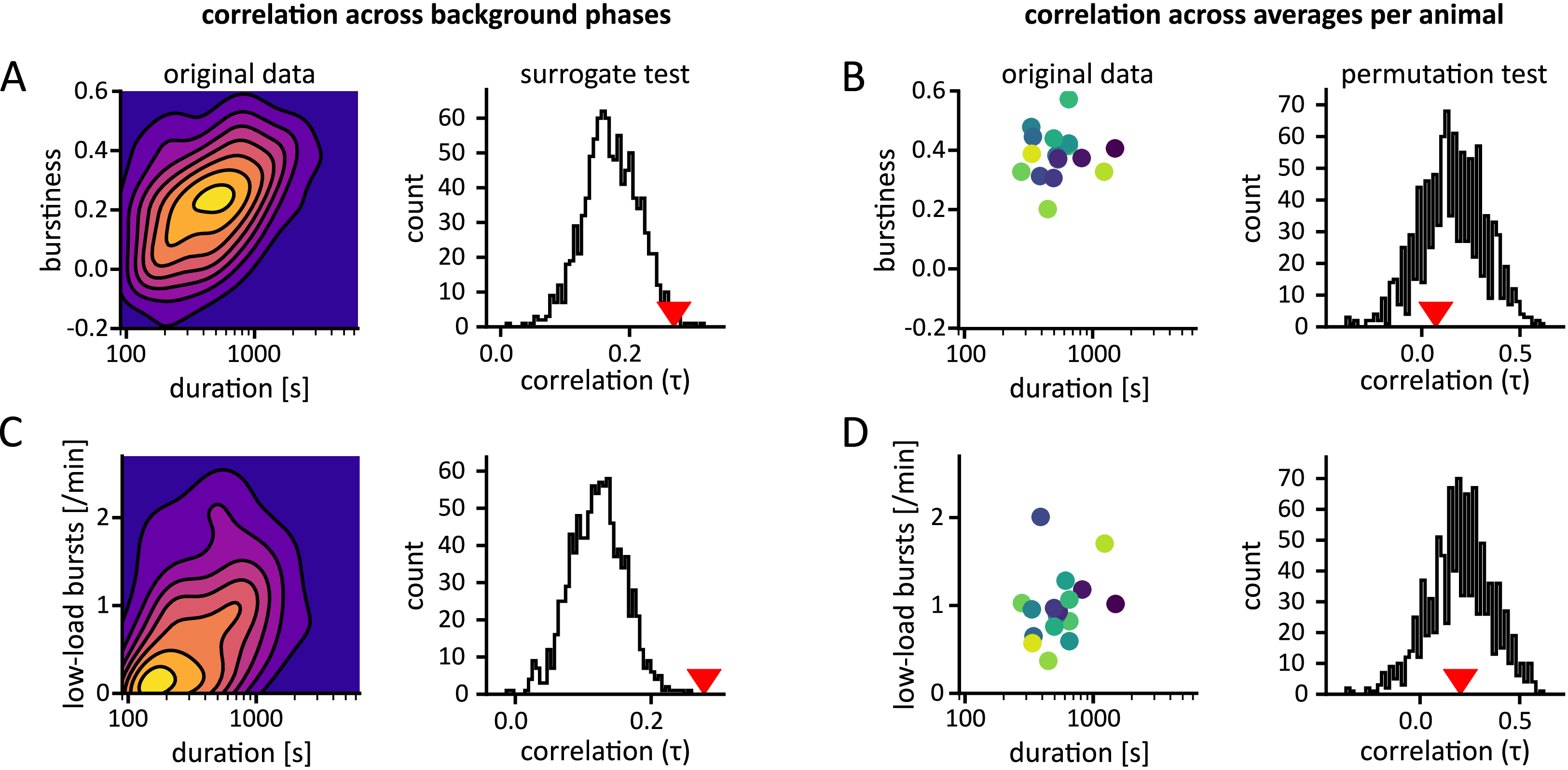

Supplement: Extended Data Figure 5-2 — Surrogate and permutation tests for correlations in Figure 5. A, Test for significance of the correlation between burstiness of spikes and the duration of background phases. The null hypothesis was that the observed correlation (left panel; repeated from Fig. 5B) was due to a continuous random process in the background generating spikes with fixed rate. Surrogate spike trains were generated from a Poissonian distribution of intervals to match the average spike rate across background phases. Surrogate correlations were obtained by computing the correlation for surrogate background phases with durations as in A, left, populated by surrogate spike trains. To assess significance, we compared Kendall’s tau of the original data (τ = 0.27, red triangle in right panel) to a distribution of Kendall’s taus obtained from 1000 surrogate correlations. psurrogate was defined as the fraction of surrogate correlations exceeding the original correlation. The burstiness of the original spikes was significantly correlated with the duration of background phases (psurrogate = 0.009). B, Using a permutation test, we assessed whether animals with on average longer background phases had spike activity with generally higher burstiness (left: original data; repeated from Fig. 5D). We generated surrogate data by randomly allocating background phases from the overall pool such that each animal was assigned as many background phases as it originally contributed to the dataset. The corresponding burstiness of a resulting surrogate animal was calculated from all interspike intervals in all background phases assigned to it. Significance was assessed by comparing the original correlation (τ = 0.07) to 1000 correlations from surrogate animals. Overall burstiness of spikes in background phases from an animal did not indicate its grand average duration of background phases (left-tailed psurrogate = 0.3). C, Significance of the correlation between the background rate of low-load bursts and the duration [file sup_enu-eN-NWR-0299-18-s09.tif]

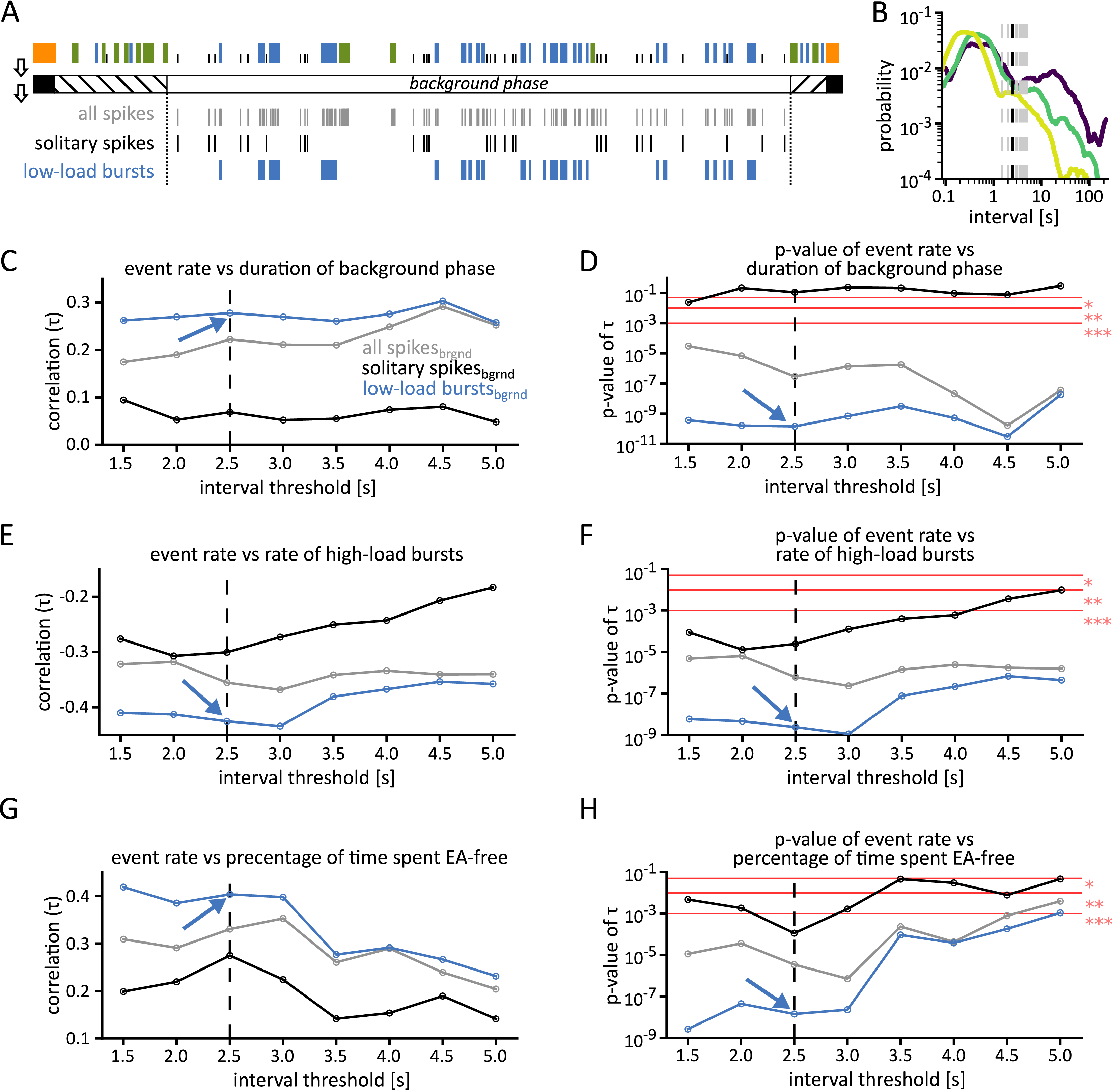

Supplement: Extended Data Figure 5-3 — Correlation effects of low-load bursts are robust against changes in burst definition. To test how robust our results were to variations in event definition, we repeated our analyses for different burst delimitation thresholds. Thus far, we had grouped spikes separated by less than 2.5 s into the same burst event. Depending on the choice of this threshold, individual spikes could be assigned to different event types and the delineation of high-load clusters, transition and background phases could change as a consequence. To test the impact of such changes in event delimitation, we varied the threshold between 1.5 and 5 s and recalculated the correlations shown in Figures 5E and 6A,D for low-load bursts, solitary spikes, and spikes in general. A, Illustration of EA in a background phase. “All spikes” comprises solitary spikes as well as those constituting low-load (blue) and medium-load bursts (green). B, Interspike interval distributions from three sessions closest to the 5th (deep purple), 50th (i.e., median, green), and 95th percentile (yellow) of total spike rate across sessions. Thresholds for burst identification were varied from 1.5 to 5 s (gray lines, black: 2.5 s corresponding to the original threshold used as detailed in Fig. 1D). C, Across all thresholds for burst delimitation, the duration of the background phase correlated most strongly with the rate of low-load bursts. Black vertical line, Interval threshold of 2.5 s. Blue arrow, Relation shown in Figure 5E. D, p values corresponding to the correlations shown in C. Red lines mark *pτ < 0.05, **pτ < 0.01, ***pτ < 0.001 significance levels. E, Of all markers, the background rate of low-load bursts was most strongly anti-correlated to the rate of high-load bursts. Blue arrow, Relation shown in Figure 6A. F, p values corresponding to the correlations shown in E. G, Across all thresholds, the background rate of low-load bursts yielded the highest correlation to the percentage of time spent without EA. Blue a [file sup_enu-eN-NWR-0299-18-s10.tif]

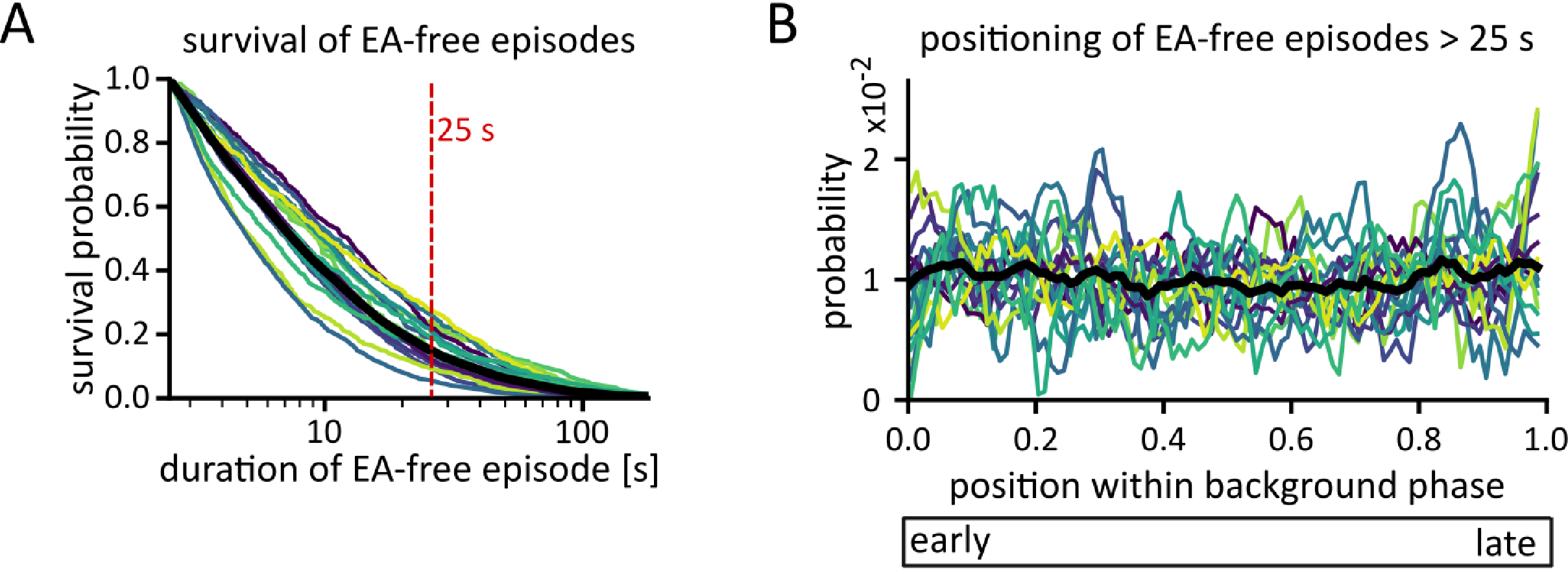

Supplement: Extended Data Figure 6-1 — Survival and positioning of EA-free episodes. A, Survival function for EA-free episodes. EA-free episodes were defined as periods within the background phase that did not contain EA events, i.e., inter-EA event intervals in the background phase. The survival function shows what fraction of EA-free episodes (y-axis) lasts for at least a certain time (x-axis). Line color indicates animal, data pooled from all animals are shown in black. The average survival function decays logarithmically up to a duration of about 20 s and then exhibits a heavy tail. B, Relative placement of long EA-free episodes (≥25 s, red line in A) within background phases. Long episodes occur throughout background phases with uniform probability (all animals: N = 2631, p = 1, χ2 testj). This indicates, that an impending change to high-load dynamics cannot readily be predicted by the presence or absence of long EA-free episodes. Download Figure 6-1, TIF file. [file sup_enu-eN-NWR-0299-18-s11.tif]

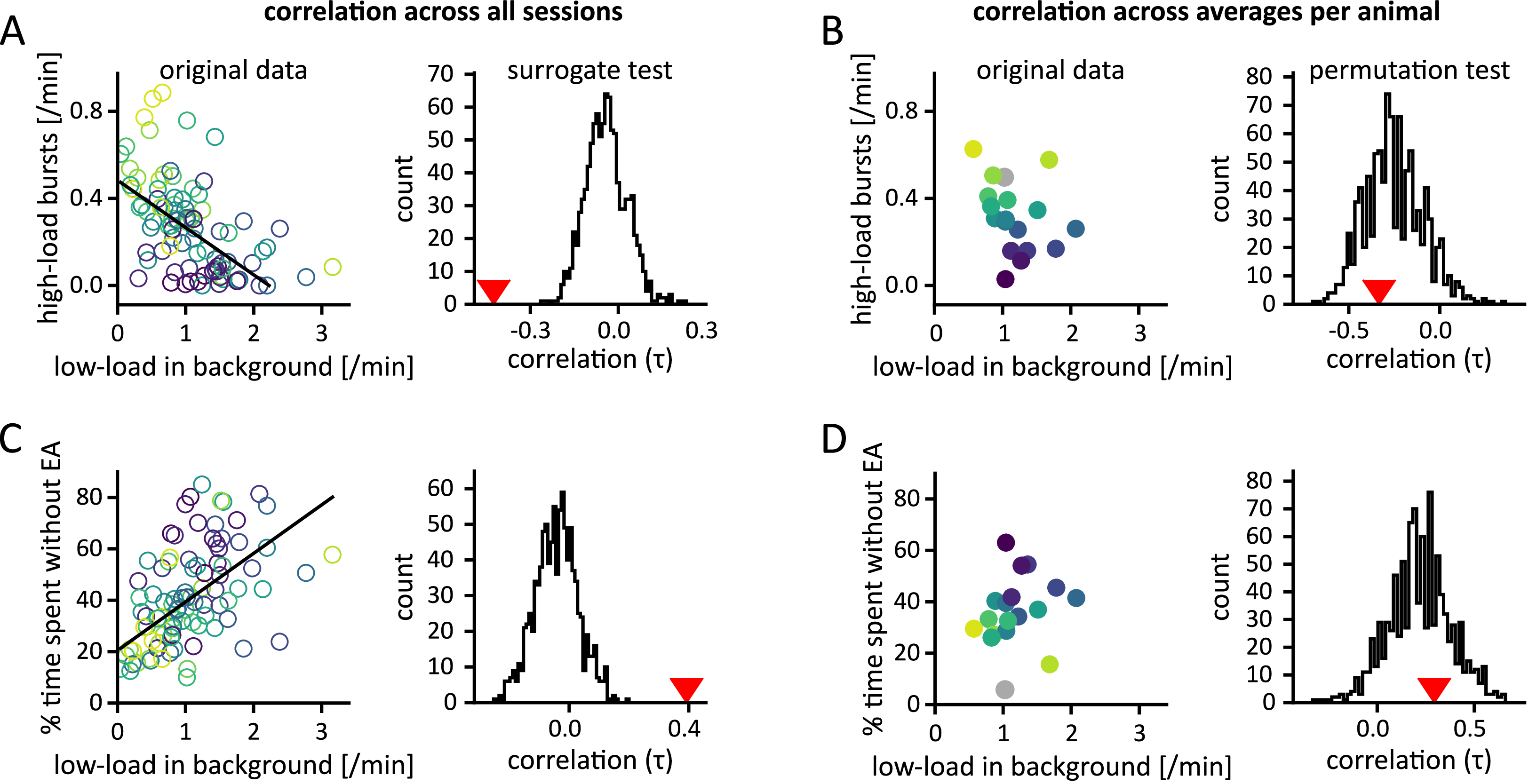

Supplement: Extended Data Figure 6-2 — Surrogate and permutation tests for correlations in Figure 6. A, Significance of the anti-correlation between background rate of low-load bursts and rate of high-load bursts across sessions (left panel; repeated from Fig. 6A). We generated surrogate background phases from time series of low-load bursts with durations drawn from the overall distribution of low-load bursts in all background phases, separated by interburst intervals drawn from a Poissonian distribution. The rate parameter of the Poissonian process was set to yield average rate of low-load bursts across all background phases. Significance was assessed by comparing Kendall’s tau of the original data (τ = –0.43, red triangle in right panel) to a distribution of Kendall’s taus obtained from 1000 surrogate correlations. psurrogate was defined as the fraction of surrogate correlations below the original correlation. The anti-correlation between background rate of low-load bursts and rate of high-load bursts was significant across sessions (psurrogate < 0.001). B, Correlation between background rate of low-load bursts and rate of high-load bursts averaged per animal (left; repeated from Fig. 6C). Using a permutation test, we assessed whether animals with on average higher background rates of low-load bursts had significantly higher or significantly lower average rates of high-load bursts. We generated surrogate data by randomly allocating sessions from the overall pool such that each animal was assigned as many sessions as it originally contributed to the dataset. Significance was assessed by comparing the original correlation (τ = –0.33) to 1000 correlations of surrogate animals. The grand average background rate of low-load bursts in an animal did not indicate its grand average rate of high-load bursts (left-tailed psurrogate = 0.31). C, Significance of the correlation between the background rate of low-load bursts and the percentage of time spent without EA (left panel; repeated from Fig. 6D). Surrogate ba [file sup_enu-eN-NWR-0299-18-s12.tif]

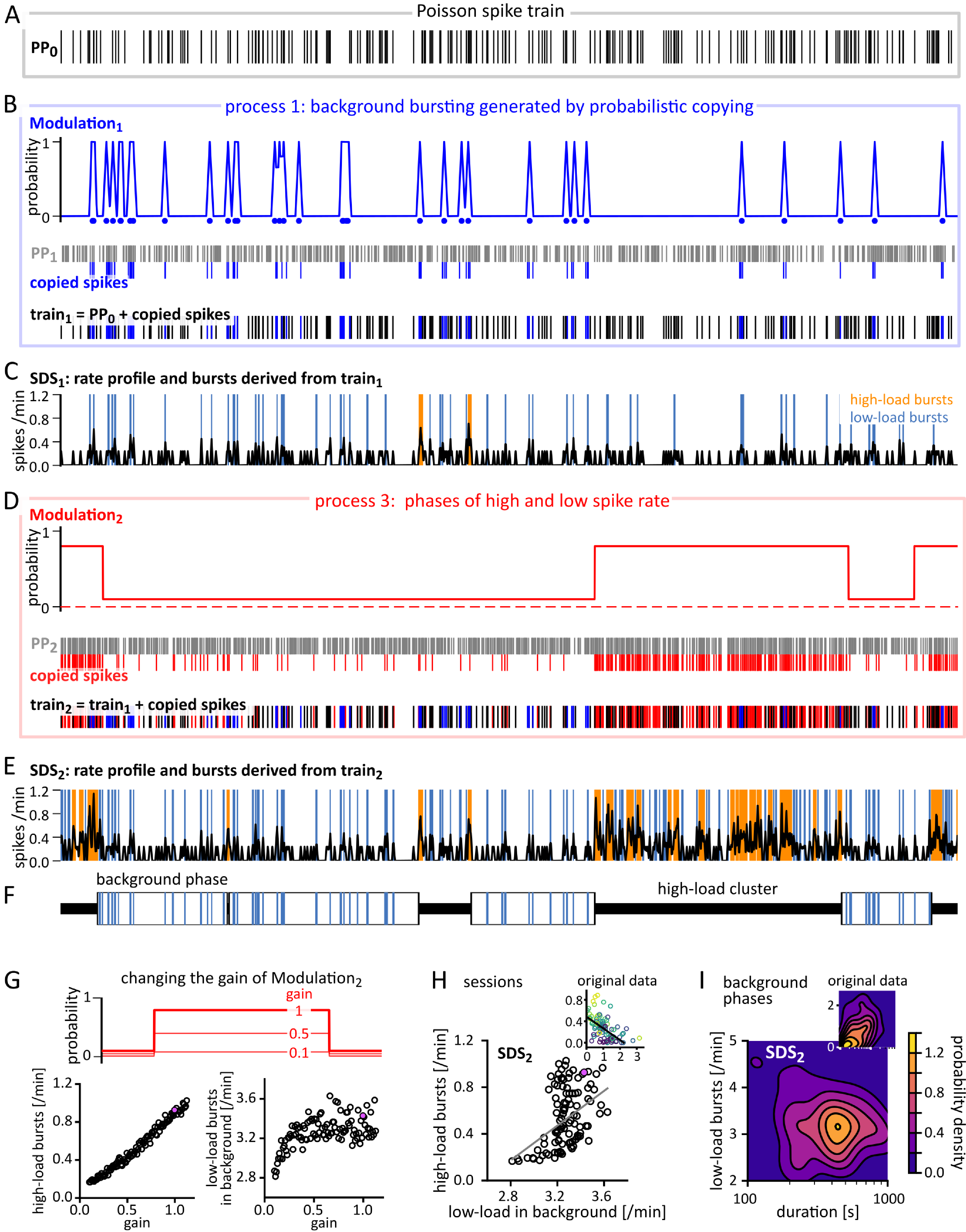

Supplement: Extended Data Figure 6-3 — Simulation of rate-modulated spike trains. To test the hypothesis that our findings could result from a rate-modulated spiking process, we simulated a surrogate dataset (SDS) of 105 sessions, each lasting 2.5 h, as nested modulated Poisson processes (PPs). Panels A–F illustrate this procedure for a 35-min segment from a surrogate session. The gain of the rate modulation was varied to simulate the range of high-load burst rates across our recording sessions. A, A first PP (PP0) was used to generate a baseline time series of spikes at a fixed rate (0.17 spikes/s). B, To generate bursts at realistic rates, we copied spikes from a second PP (PP1; fixed rate 0.28 spikes/s) with a probability given by the modulation function Modulation1 to create train1. Peaks in Modulation1 were timed randomly with a separate PP (dots in B) according to the mean rate of bursts across all sessions (0.92 bursts/min). This time series was convolved with a triangular kernel to create a probability density function for copying spikes. C, Bursts in train1 were detected as in the original recordings (spikes closer than 2.5 s were grouped into the same burst; Fig. 1D). To assign bursts to low-load and high-load classes, we could not use our original classifier, because the within-burst structure was not replicated. We therefore used the area under the curve (AUC) of the rate profile (black) of each burst to approximate spike load (compare Extended Data Fig. 1-1). From the AUC values we then defined a threshold to separate high-load from low-load burst. The threshold was adjusted to reproduce in SDS2 (see E) the 3:1 ratio of the rates of low-load to high-load bursts observed in our recordings, i.e., bursts with AUC values above the 76th percentile were considered high-load bursts, the others were low-load bursts. Note that we neglected medium-load bursts for simplicity. Most but not all bursts in SDS1 coincided with peaks in Modulation1 with only few qualifying as high-load bursts. D, To reproduc [file sup_enu-eN-NWR-0299-18-s13.tif]
